# Tunable microsecond dynamics of an allosteric switch regulate the activity of a AAA+ disaggregation machine

**DOI:** 10.1038/s41467-019-09474-6

**Published:** 2019-03-29

**Authors:** Hisham Mazal, Marija Iljina, Yoav Barak, Nadav Elad, Rina Rosenzweig, Pierre Goloubinoff, Inbal Riven, Gilad Haran

**Affiliations:** 1 Department of Chemical and Biological Physics, Weizmann Institute of Science, 761001 Rehovot, Israel; 2 Department of Chemical Research Support, Weizmann Institute of Science, 761001 Rehovot, Israel; 3 Department of Structural Biology, Weizmann Institute of Science, 761001 Rehovot, Israel; 4 Department of Plant Molecular Biology, Faculty of Biology and Medicine, University of Lausanne, CH-1015 Lausanne, Switzerland

## Abstract

Large protein machines are tightly regulated through allosteric communication channels. Here we demonstrate the involvement of ultrafast conformational dynamics in allosteric regulation of ClpB, a hexameric AAA+ machine that rescues aggregated proteins. Each subunit of ClpB contains a unique coiled-coil structure, the middle domain (M domain), proposed as a control element that binds the co-chaperone DnaK. Using single-molecule FRET spectroscopy, we probe the M domain during the chaperone cycle and find it to jump on the microsecond time scale between two states, whose structures are determined. The M-domain jumps are much faster than the overall activity of ClpB, making it an effectively continuous, tunable switch. Indeed, a series of allosteric interactions are found to modulate the dynamics, including binding of nucleotides, DnaK and protein substrates. This mode of dynamic control enables fast cellular adaptation and may be a general mechanism for the regulation of cellular machineries.

## Abstract (web summary)

Large protein machines are tightly regulated through allosteric communication channels. Here authors use single-molecule FRET and demonstrate the involvement of ultrafast conformational dynamics in the allosteric regulation of ClpB, a hexameric AAA+ machine that rescues aggregated proteins.

## Introduction

Biomolecular machines convert chemical energy into work, following a series of conformational transitions^1,2^. The activity of these machines, which participate in multiple cellular processes^3,4^, is highly regulated, often through ligand binding^5,6^. The interaction of ligands with a protein machine may initiate conformational changes far away from the binding site. This phenomenon has been termed allostery by Monod, Jacob and coworkers^7,8^, and studied extensively over the years^9,10^. While the original work emphasized the thermodynamic states of the transforming proteins, Dryden and Cooper proposed that allostery can also involve changes in conformational dynamics^11^. In recent years, there has been much interest in defining the potential role of dynamics in allosteric transitions^12–17^. Particularly intriguing are large-scale correlated motions of domains and subunits^18–21^. It is often the case that, while the end points of such correlated motions are structurally well-characterized, the transitions between them have not been directly observed^22^.

ClpB is a homohexameric protein machine from bacteria that belongs to the Hsp100 family of ATPases associated with diverse cellular activities (AAA+). ClpB and its yeast homolog, Hsp104, are involved in rescuing proteins from aggregates^23–25^. This activity is performed in conjunction with several co-chaperones, the most important of which are bacterial Hsp70 (DnaK) and its co-chaperones DnaJ and GrpE^25^. Each subunit of ClpB is built of four domains: the N-terminal domain, two nucleotide binding domains (NBD1 and NBD2), and the coiled-coil middle domain (M domain), which is inserted in NBD1 and positioned at the outer surface of the ClpB complex^26–28^. It has been suggested that the M domain acts as a regulatory switch that toggles between an active conformation, in which it can bind DnaK and thereby activate the ClpB disaggregation function, and an inactive conformation, in which it does not bind DnaK, repressing the ClpB disaggregating machine^27,29–31^. The ClpB-DnaK complex uses the energy of ATP to destabilize and convert protein aggregates into native proteins. Remarkably, a close ortholog of ClpB in *E. coli*, named ClpA, totally lacks the M domain and, therefore, does not seem to interact with DnaK^32^. Several studies indicated that the M domain is flexible and mobile and may adopt multiple conformations^26,33–38^. Significantly, it was proposed that this mobility is important for disaggregation function^26,35^. However, there is no real-time measurement of the function-related motions of the M domain and how they are regulated by nucleotides and cofactors.

Here we apply single-molecule FRET (smFRET) spectroscopy to probe M-domain motions in *Thermus thermophilus* (*TT*) ClpB. We study double-labeled protein molecules diffusing in solution, and use a recently developed maximum likelihood algorithm^39^ to extract information on the states of the M domain and their interconversion rates. We identify and structurally characterize the active and inactive conformations of the M domain. We also find that M-domain motions take place on the microsecond time scale, that ATP binding to NBD1 and NBD2 exerts a modulating allosteric effect on these motions, and that DnaK or protein substrate binding enhance them significantly. Most importantly, we establish the M domain as a tunable allosteric switch, which modulates the activity of the machine based on the ratio between its active and inactive conformations.

## Results

### Ultrafast dynamics of the ClpB M domain

**Fig. 1:**
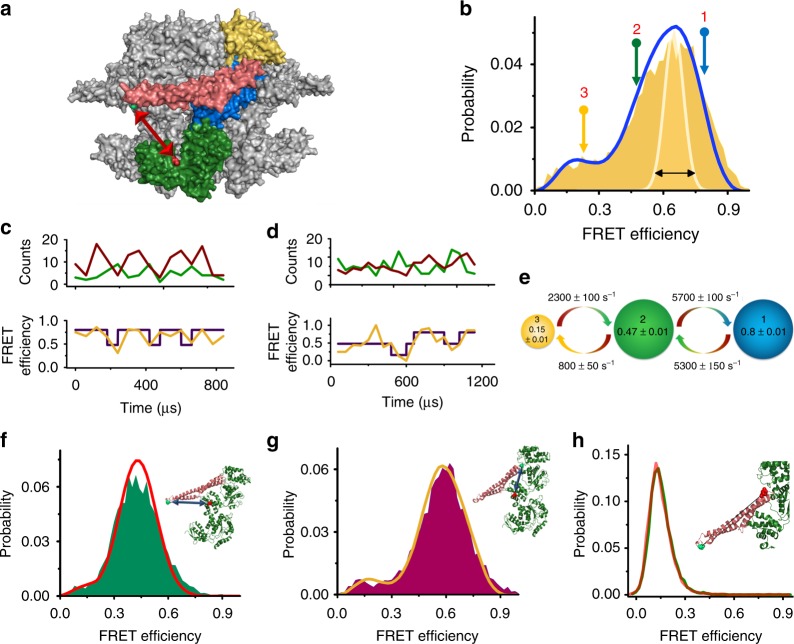
M-domain conformational dynamics measured with smFRET. **a** Hexameric model of ClpB reconstructed from the *Thermus thermophilus* monomer structure (PDB: 1QVR)^26,53^. One monomer is highlighted, with the N-terminal domain in gold, the two nucleotide binding domains, NBD1 and NBD2, in blue and green, respectively, and the M domain in salmon color. The red arrow points to the attachment positions for donor and acceptor dyes (S428 and S771, respectively). **b** FRET efficiency histogram of double-labeled ClpB (S428C-S771C). White line and black arrow indicate expected width based on shot noise. The blue line is the outcome of a ”recoloring” simulation (see Methods). The arrows above the histogram show the positions of the three states obtained from H^2^MM analysis. **c**, **d** Two single-molecule trajectories binned at 60 µs. Top panel in each section shows the donor and acceptor signals (green and red, respectively), while the bottom panel shows the FRET efficiency obtained from the signals in orange, with the purple line representing state assignment obtained from H^2^MM analysis. **e** Results of the H^2^MM analysis of a smFRET measurement of ClpB (S428C-S771C) in the presence of ATP. See also Supplementary Fig. 8. The interconversion rates between the states are shown above and below each arrow. The occupancy of each state is represented by the size of the circle (state 1–43 ± 1%, state 2–42 ± 1%, and state 3–15 ± 1%). FRET efficiencies are shown inside the circles. **f**, **g** FRET efficiency histograms from experiments with two additional donor-acceptor pairs: 428–359 (**f**) and 483–359 (**g**). Inset in each panel shows the monomer structure with the donor and acceptor sites marked (green and red, respectively). Red and orange lines in (**f**) and (**g**), respectively, are the outcomes of “recoloring” simulations (see also Supplementary Fig. 9). **h** FRET efficiency histograms of a ClpB variant with cysteines on the two ends of the M domain, S428C-N487C (see inset for structure, with cysteine positions shown in green and red). Histograms were measured in the presence of 2 mM ATP (red) and in the absence of ATP (green). The Errors represent standard errors of the mean. Source data are provided as a Source Data file

We designed a series of double-cysteine mutants that would allow us to probe the conformational changes of the M domain using smFRET spectroscopy^27,40,41^. First, we located one probe on the so-called motif 1 of the M domain (on residue 428) and the second probe on NBD2 (residue 771) (Fig. 1a). We labeled this construct with donor and acceptor fluorescent dyes. To ensure that we measure FRET efficiency from a single protomer of each hexameric complex, we assembled ClpB hexamers using a ratio of unlabeled to double-labeled subunits of 100:1 (see Methods and Supplementary Figs. 1–5 for ClpB purification, assembly, labeling and other control experiments).

The conformational dynamics of labeled molecules were measured in solution in the presence of 2 mM ATP. Under these conditions, ClpB hexamers were well assembled (see Supplementary Information section “Validating the structural integrity of ClpB hexamers” and Supplementary Figure 3 for a discussion on the stability of our hexamers). Freely diffusing molecules of ClpB emitted bursts of photons as they passed through a focused laser beam. The arrival time and the color of each photon were registered in the donor and acceptor channels. A FRET efficiency histogram was constructed from molecules that contained both donor and acceptor dyes (see Supplementary Figure 6 for filtration of molecules) and showed a major population at a FRET efficiency of 0.65 ± 0.01, and a minor population at a FRET efficiency of 0.23 ± 0.01. Importantly, the major peak was found to be much broader than expected based on shot noise (Fig. 1b), an indication of two or more conformations under fast exchange dynamics^41,42^. Inspection of binned photon trajectories (Fig. 1c, d and Supplementary Fig. 7), which showed FRET efficiency fluctuations, reinforced this observation.

To extract information on fast dynamics of the M domain we turned to H^2^MM, a powerful photon-by-photon hidden Markov model algorithm developed recently in our lab^21,41^. We found that the minimal number of states required to fit single-molecule trajectories is three: two major populations at FRET efficiencies of 0.8 ± 0.01 (state 1) and 0.47 ± 0.01 (state 2), and a minor population at a FRET efficiency of 0.15 ± 0.01 (state 3). The relative populations of these states were 0.43 ± 0.01, 0.42 ± 0.01, and 0.15 ± 0.01, respectively. Interestingly, we found that these three states are exchanging in a sequential manner (Supplementary Fig. 8), with fast transition rates between state 1 and 2, *k*_12_ = 5300 ± 150 s^−1^ and *k*_21_ = 5700 ± 100 s^−1^, and slower transition rates between states 2 and 3, *k*_23_ = 800 ± 50 s^−1^ and *k*_32_ = 2300 ± 100 s^−1^ (Fig. 1b–e, Supplementary Table 1). We validated the H^2^MM analysis using four different methods, including stochastic recoloring of the data^21,43^ (Fig. 1b, Supplementary Fig. 9A), segmentation analysis^21^, dwell time distribution analysis, and cross-correlation analysis (Supplementary Fig. 10A, E, I and Supplementary Table 2). An indication of the correct modeling of the data was that for all mutants and conditions we used (independent measurements), we obtained essentially the same FRET values for the three states (see Source data file). We also tested explicitly that a model with discrete states is the best way to describe the data, as discussed in the Supplementary Information section “Testing the suitability of the three-state model”. We repeated the experiment in the presence of the slowly hydrolyzable nucleotide ATPγS instead of ATP, with very similar results (Supplementary Fig. 11). This result indicates that nucleotide hydrolysis does not directly govern M-domain dynamics, which is commensurate with the fact that the rates of the M-domain conformational changes were much higher than ATP hydrolysis rate (3.2 ± 0.1 min^−1^, Supplementary Fig. 11 and Supplementary Fig. 4A).

To further characterize M-domain dynamics, we generated two additional FRET pairs: the pair S428C-S359C to probe conformational changes of motif 1 (Fig. 1f), and the pair Q483C-S359C to probe motif 2 (Fig. 1g). FRET histograms of both mutants also showed a major broad peak and a minor peak (Fig. 1f, g, Supplementary Fig. 9B, C), and H^2^MM analysis showed that they were also best described with a three-state model. The transition rates between the two populations in the major peak were similar to those observed with the FRET pair S428C–S771C (Supplementary Table 1). To exclude the possibility that the observed fast dynamics are due to conformational changes within the M domain, such as unfolding of motif 2^26,35,37^, we labeled the M domain itself, with one dye on motif 1 (S428C) and the second on motif 2 (N487C). The FRET histogram of this variant showed a single narrow peak, both in the presence of 2 mM ATP and in its absence (Fig. 1h). This result demonstrates very clearly that the M domain moves as a rigid body.

The above findings suggest that the M domain is highly dynamic and interconverts between two major states and a third minor state much faster than ClpB hydrolyzes ATP or disaggregates proteins (Supplementary Fig. 4A and Supplementary Fig. 5D). This may explain why many structural studies based on cryo-electron microscopy (Cryo-EM) could not resolve the complete structure of the M domain, which was absent or only partially observed in electron density maps due to its high mobility^36,44,45^. In this study, we focus on analysis of the two major dynamic states of the M domain, states 1 and 2, and their relation to various perturbations exerted on ClpB. The third and minor state of the M domain will be discussed in a future publication. We start here by generating a structural model for states 1 and 2, based on the FRET efficiency values obtained with the three FRET pairs.

### A structural model for M-domain conformations

**Fig. 2:**
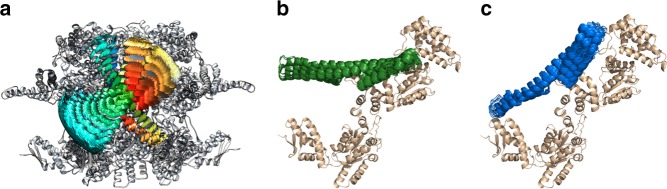
Obtaining a structural model for the M-domain conformations. **a** A cartoon showing all allowed conformations of the M domain (shown in rainbow color), superimposed on the hexamer structure (gray), which is modeled on the basis of the crystal structure of the ClpB monomer^53^. **b**, **c** M-domain conformation in state 2 ((**b**), shown in green), and state 1 ((**c**), shown in blue). Shown are the 10 conformations with the lowest chi-squared values as obtained from our calculation (see Methods). The predicted FRET efficiency values corresponding to each structure were calculated taking the dye linkers into account^49,50^. See Supplementary Table 3 for further analysis of the best structures and Methods for the calculation procedure. Source data are provided as a Source Data file

To obtain structural information for the two main states of the M domain, we used the three sets of FRET efficiencies obtained from smFRET experiments with the three FRET pairs (Supplementary Table 3), combined with a geometrical triangulation approach. Triangulation based on several FRET pairs has been applied to obtain structural models of protein complexes^46–52^. Here we developed a somewhat different analysis scheme from these works. Starting with the hexameric structural model of *TT* ClpB^53^, which is based on the subunit crystal structure^26^, we produced multiple conformations of the M domain by rotating it as a rigid body around its connection point to NBD1 (residue 396). A set of ~16,000 conformations was initially generated, and we then excluded all conformations that caused a steric clash within the same protomer and the adjacent protomers (see Methods), which left us with only ~5% of the whole set (Fig. 2a). We then calculated the FRET efficiency values for the three FRET pair locations (S428C-S771C, S359C-S428C, and Q483C-S359C) in each of the allowed conformations, taking dye and linker conformations into account (see Methods and Supplementary Notes), and used them to calculate a “chi-squared” value for each conformation. Two sets of chi-squared values were calculated, one for state 1 and one for state 2. We then selected a group of ten structures with the lowest chi-squared values (Fig. 2b, c, Supplementary Table 3). This procedure generated structural models for the M domain in each of the states (Fig. 2b, c).

Strikingly, the conformation of the M domain in state 2 is similar to the conformation of the domain observed in the ClpB crystal structure^26^ (Fig. 2b, Supplementary Table 3), and in a recent cryo-EM study^27^. The axis of the M domain in state 2 is essentially perpendicular to the axis of symmetry of ClpB, so that all six M domains are parallel to each other. Therefore, state 2 can be identified with the inactive state of the M domain as it was characterized previously from mutant analysis and cryo-EM studies^29,31^. A quantitative analysis of the structural models of state 2 of M-domain showed that the best model had a root mean squared deviation (RMSD) of 1.2 Å from the crystal structure, while all best ten models had an average RMSD value of 5.5 ± 3.1 Å (see Supplementary Table 3 and Supplementary Fig. 12A, B for comparison with the recent high resolution cryo-EM structure^27^).

State 1 of the M domain is tilted from the parallel, inactive conformation by 40 ± 2° (Fig. 2c, Supplementary Table 3), and motif 2 is exposed and accessible for DnaK interaction^30^. This structure therefore corresponds to the active state. It is important to note that there has been no previous complete and accurate conformational model for the active state of the M domain, likely due to its high mobility^27,31,54^, though an estimate for the structure of motif 1 was provided by Deville et al.^27^ (see Supplementary Fig. 12C for a comparison with this structure). This work presents a complete and accurate model for the M domain in this state. In our suggested model, motif 1 tilts by moving away from NBD1 and towards NBD2 in the active state. In the tilted conformation, motif 1 remains on the same level with NBD1 of the adjacent protomer (Supplementary Fig. 12D), in good agreement with the orientation previously proposed by Deville et al^27^.

### Allosteric interactions of the M domain and the NBDs

**Fig. 3:**
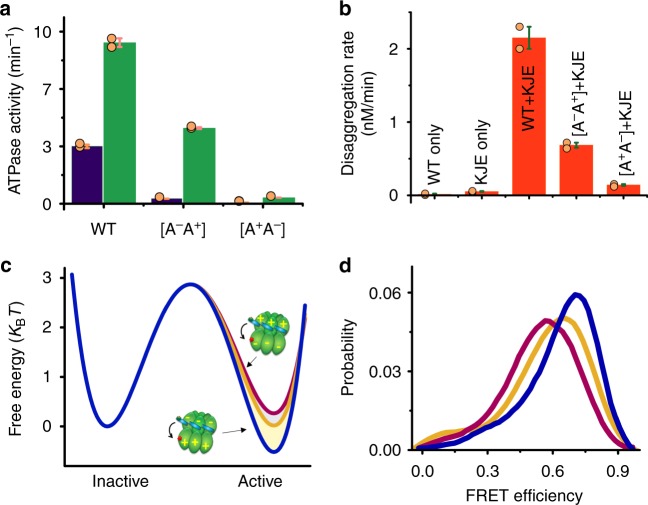
Nucleotide binding sites affect M-domain dynamics. **a** Basal and κ-casein stimulated ATPase activities (in purple and green, respectively) of the WT ClpB and the two nucleotide binding site mutants, [A^−^A^+^] and [A^+^A^−^]. The WT and [A^−^A^+^] activities were enhanced significantly by κ-casein. **b** Disaggregation activity of ClpB WT and Walker A mutants. [A^−^A^+^] retains a significant level of disaggregation activity, while [A^+^A^−^] is less active. **c** Free-energy profiles of the M domain calculated from H^2^MM populations and rates as obtained from analysis of smFRET data of the WT (orange) and of [A^−^A^+^] (blue) and [A^+^A^−^] (purple). Barrier heights in this and other figures were calculated using the Arrhenius equation with a pre-exponential factor of 10^5^ s^−1^. Transition rates (in s^−1^) are given in Supplementary Table 4. State 1 of [A^+^A^−^] is destabilized compared to the WT, and the transition rate from state 1 to state 2 is increased, while state 1 of [A^−^A^+^] is stabilized, so that the transition rate from state 1 to state 2 is decreased (for the values of the ratio between states see Supplementary Table 5). **d** FRET efficiency histograms of the WT (orange), [A^+^A^−^] (purple), and [A^−^A^+^] (blue). The shifts of the [A^−^A^+^] and [A^+^A^−^] histograms to higher and lower FRET efficiency, respectively, reflect the stabilization or destabilization of state 1. Orange circles in (**a**) and (**b**) represent separate repeats of the measurements. Error bars represent standard errors of the mean. Source data are provided as a Source Data file

The binding and hydrolysis of ATP have been shown to affect the activity of ClpB in distinct ways^28,36,55–59^. Each of the two nucleotide binding sites, NBD1 and NBD2, contains conserved Walker A and Walker B motifs^60,61^. Mutation of the Walker A motif abolishes ATP binding, while mutation in Walker B does not affect binding but prevents hydrolysis. To understand allosteric interactions between the ATP binding sites and the M domain, we tested the effect of Walker motif mutations on M-domain dynamics. We started by abolishing ATP binding to NBD1 using the mutation K204T in its Walker A motif^61–63^. This variant (which we designated [A^−^A^+^]) was found to be well assembled (Supplementary Fig. 3 and Supplementary Fig. 13), as previously reported for other similar Walker A mutants^29,64^, showed prominent disaggregation activity but only weak ATPase activity, which was enhanced upon binding to the substrate κ-casein (Fig. 3a, b and Supplementary Fig. 14). smFRET studies of [A^−^A^+^] showed a reduced transition rate from state 1 to state 2 compared to the WT, *k*_12_ = 3500 ± 100 s^−1^, but a similar transition rate in the reverse direction, *k*_21_ = 5800 ± 350 s^−1^ (Fig. 3c, Supplementary Fig. 7B, C, Supplementary Fig. 9D and Supplementary Table 2 and Supplementary Table 4). This change in transition rates led to an increase in the active/inactive state ratio, from 1.00 ± 0.01 in the WT to 1.63 ± 0.02 in [A^−^A^+^] (Supplementary Table 5). We constructed an approximate free-energy profile from these parameters, which is compared to the corresponding free-energy profile of the WT in Fig. 3c. The free-energy barrier heights in these profiles and additional ones presented in the paper were calculated using the Arrhenius equation, with a conservative choice of 10^5^ s^−1^ for the pre-exponential factor. Theoretical^65^ and experimental^66^ estimates suggest that this factor might in fact be larger than 10^6^ s^−1^. The increase in the population ratio of the two states was reflected in the FRET efficiency histogram of this mutant, which showed a shift to higher FRET efficiency values relative to the WT (Fig. 3d).

We then abolished ATP binding to NBD2 by introducing a K601T mutation^61^. This variant (termed [A^+^A^−^]) was also found to be well assembled (Supplementary Fig. 3 and Supplementary Fig. 13)^29,64^, but in contrast to [A^−^A^+^] its very weak ATPase activity was not enhanced by κ-casein binding and its disaggregation rate was lower (Fig. 3a, b and Supplementary Fig. 14). smFRET studies of this mutant showed an increased transition rate from state 1 to state 2, *k*_12_ = 7700 ± 100 s^−1^, and again a similar inverse rate, *k*_21_ = 6000 ± 200 s^−1^ (Fig. 3c, Supplementary Fig. 9E and Supplementary Table 4). The population ratio between the states decreased from 1.00 ± 0.01 to 0.75 ± 0.02 (Supplementary Table 5).

Here we found both Walker A mutants to be well assembled under our experimental conditions, which suggests that both NBDs play a role in hexamer stabilization (Supplementary Fig. 3). However, we clearly observe that NBD2 contributes more significantly to the machine activity (Fig. 3a, b). Our results suggest a significant allosteric effect of nucleotide binding on M-domain dynamics. Nucleotide binding to NBD2 stabilizes the M domain in the active state whereas nucleotide binding to NBD1 stabilizes the inactive state. Coupling of the M-domain structural changes to nucleotide binding to the NBDs has been observed^28,29,33–35,45,67^, most recently in a study by Sugita et al.^68^, who reported that ATP binding to NBD1 shifts the M-domain to a tilted conformation. Here we show that this coupling is exerted through an effect on M-domain dynamics. In particular, the transition rate from state 2 to state 1 is not significantly affected by the mutations whereas the transition rate form state 1 to state 2 is modulated. The regulation of the M-domain dynamics is thus exerted through state 1, namely the active conformation. Together, the two NBDs exert a moderating influence on M-domain dynamics and conformation. At a relatively low ATP concentration, where NBD2 binds the nucleotide better (with a K_0.5_ of 320 ± 20 µM, see Supplementary Table 6), the M domain is preferentially in its active state, while at a higher ATP concentration NBD1 also binds ATP well (with a K_0.5_ of 500 ± 73 µM, see Supplementary Table 6), pushing the M domain to the inactive state. Indeed, we found that, due to these two contrasting effects, at saturating ATP concentrations the M domain in WT ClpB is ~50% of the time in each of the states (Fig. 1b). The results described above with the [A^−^A^+^] and [A^+^A^−^] variants could not be attributed to structural perturbations caused by the mutations, but rather reflected the lack of ATP binding to one of the nucleotide binding sites. To prove this, we constructed the double Walker B mutant [B^−^B^−^] (E271A, E668A), which could bind but not hydrolyze ATP in both NBDs. This mutant was found to be well assembled (Supplementary Fig. 13), but as expected^55,69^, no ATPase activity and therefore no disaggregation activity was observed (Supplementary Fig. 15A–C). However, smFRET measurements of this mutant showed M-domain dynamics resembling the WT, mirroring the already-mentioned measurements with the non-hydrolyzable ATPγS (Supplementary Fig. 15A, Supplementary Fig. 11 and Supplementary Tables 4-5).

### DnaK and substrate binding regulate M domain dynamics

**Fig. 4:**
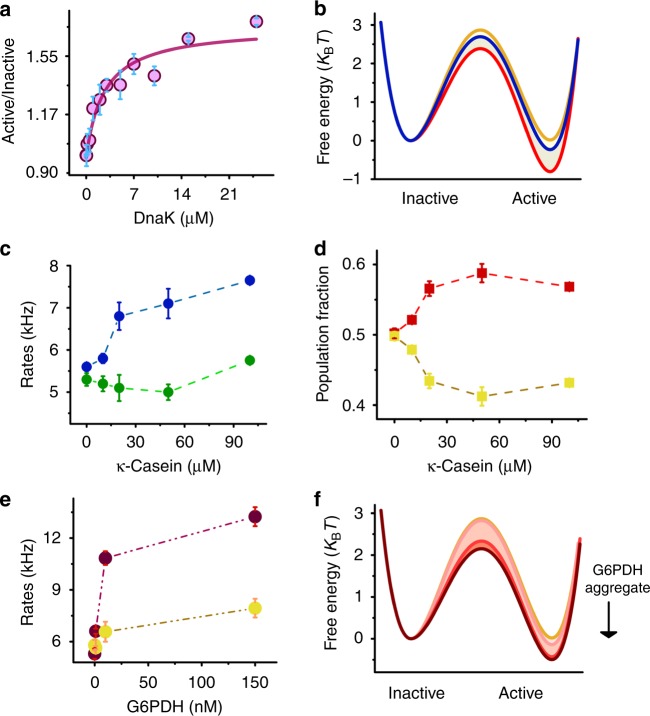
DnaK and substrate binding stabilize the active state of the M domain. **a** Population ratio of state 1 and state 2 (active/inactive) shows a monotonic increase (purple circles). The solid line is a fit to a binding model, yielding a *K*_d_ of 3 ± 1 µM. **b** Free-energy profiles of the M domain calculated from H^2^MM-derived populations and rates obtained from the analysis of smFRET data at three concentrations of DnaK: 0 µM (orange), 5 µM (blue) and 25 µM (red). Both rates increase with DnaK, but *k*_21_ increases more, leading to the accumulation of the active state. For the values of the rates see Supplementary Table 7. **c** ClpB was incubated with increasing concentrations of κ-casein. Measurements showed the rate *k*_21_ is increasing as a function of κ-casein (blue color), while the rate *k*_12_ is decreasing (green color) (Supplementary Table 8). **d** The active (red) and inactive (yellow) state populations as a function of κ-casein concentration are shown, demonstrating an anti-correlated trend. **e** ClpB was incubated with increasing concentrations of G6PDH aggregates (treated with 10 µM DnaK and 1 µM DnaJ). smFRET measurements and H^2^MM analysis showed that *k*_21_ dramatically increased with increasing G6PDH aggregate concentration (purple color), while *k*_12_ increased only slightly (yellow) (Supplementary Table 9). The dashed lines in C-E are intended to guide the eye only. **f** Free-energy profiles calculated from the rates and the model as obtained from a smFRET measurement and H^2^MM analysis of ClpB in the presence of G6PDH aggregates. By increasing the G6PDH aggregate concentration (light red to dark red color) state 1 is stabilized and the free-energy barrier between state 1 and 2 is reduced (for the ratio between the states see Supplementary Table 10), affecting mostly the transition from state 2 to state 1. Error bars represent standard errors of the mean. Source data are provided as a Source Data file

DnaK is the main component of the co-chaperone system in the disaggregation process, which has been shown to act both upstream and downstream of ClpB^25,30,63,70^. In addition, DnaK binds to the M domain at motif 2^63^, regulating its disaggregation activity. We conducted smFRET measurements of ClpB in the presence of increasing concentrations of DnaK (see Supplementary Fig. 1^6^ for DnaK characterization). At all concentrations, a fast exchange between states 1 and 2 was observed (Supplementary Fig. 7D, E, Supplementary Fig. 9F, Supplementary Fig. 10B, F, J, Supplementary Table 2 and Supplementary Table 7). However, the population ratio between state 1 and state 2 was found to increase with the co-chaperone concentration, from a value of 1.00 ± 0.01 in its absence to a value of 1.85 ± 0.03 at 25 µM (Fig. 4a). This change in population ratio was reflected also in the free-energy profile (Fig. 4b). Remarkably, the population of the third and minor state of the M domain reduced dramatically as the concentration of DnaK increased (Supplementary Fig. 17).

A fit of the data in Fig. 4a to a simple isothermal binding function retrieved a dissociation constant of 3 ± 1 µM, which agrees well with literature values^63,71,72^. Interestingly, the disaggregation activity of ClpB also increased with DnaK concentration in a manner characterized by a similar *K*_d_ (Supplementary Fig. 18).

These results demonstrate that DnaK binding does not simply stabilize the M domain in its active state, as previously suggested^29,30^, but rather changes the dynamic equilibrium between the active and inactive states in favor of the former. This is surprising, since the association and dissociation rates of DnaK are expected to be significantly smaller than the measured exchange rate (at saturation *k*_12_ is 4300 ± 200 and *k*_21_ is 9100 ± 350, see Supplementary Fig. 9F, Supplementary Fig. 10B, F, J, Supplementary Table 2 and Supplementary Table 7). Further, it has been proposed that DnaK cannot bind to the inactive state, due to interactions of the tail of each M domain with the head of its neighbor. To reconcile this seeming discrepancy, we note that the FRET efficiency of the inactive state slightly increased in the presence of saturating DnaK concentrations (from 0.47 ± 0.01 to 0.50 ± 0.01), possibly indicating that the DnaK-bound M domain does not attain the fully parallel conformation characteristic of the inactive state in the unbound protein.

Two recent cryo-EM studies of ClpB are in dispute with each other regarding the effect of substrate binding to ClpB on the M domain. Deville et al. reported that the M domain adopts a more inactive, horizontal structure upon substrate binding to ClpB in the presence of ATPγS^27^. Gates et al., on the other hand, proposed that M-domain conformational changes propagate around the hexamer in accordance with the nucleotide binding state of each protomer^28^. Since our own results demonstrate that the M domain conformational changes are much faster than other steps in the ClpB cycle, we wondered if substrate binding to ClpB might lock the M domain in one of its states. We first studied M domain dynamics in the presence of the soluble substrate κ-casein^73,74^. ClpB was incubated with 2 mM ATP and 10–100 µM κ-casein (a concentration range that led to a significant enhancement in the ATPase activity^74–77^, Fig. 3a and Supplementary Fig. 4C). smFRET experiments showed that while *k*_12_ decreased as a function of κ-casein concentration, *k*_21_ increased, leading to the accumulation of active state population (Fig. 4c, d, Supplementary Fig. 7F, G, Supplementary Fig. 9G, Supplementary Fig. 10C, G, K, Supplementary Table 2 and Supplementary Table 8). At the same time, the ATPase activity of ClpB increased significantly (Fig. 3a and Supplementary Fig. 4C). This enhancement is likely due to the relative increase of the M domain active state population. The effect of M-domain dynamics on ClpB activity will be further discussed in the next section.

Interestingly, similar M-domain dynamics were observed when ATP was replaced with the non-hydrolyzable variant ATPγS (Supplementary Fig. 19). These results are in better agreement with the observations of Gates et al.^28^, who reported two conformations of the M domain under similar conditions, than with those of Deville et al.^27^. We also tested the effect of an aggregated substrate, using glucose-6-phosphate dehydrogenase (G6PDH) as a model^78,79^. A volume of 900 nM of G6PDH aggregates (see Methods) was mixed with 10 µM DnaK, 1 µM DnaJ, and 100 µM ATP to form complexes^78–80^. The effect of different concentrations of these complexes on M-domain dynamics was tested. A dramatic increase of both *k*_12_ and *k*_21_ was observed (Fig. 4e, Supplementary Table 9). However, *k*_21_ increased more significantly than *k*_12_, leading to a rise of the active state population (Fig. 4f and Supplementary Table 10), similar to the effect of κ-casein. It is important to note that the substantial increase of M-domain dynamics is unlikely to be due to free DnaK binding, as the latter was present here at a low concentration, though we cannot rule out that aggregate binding to DnaK increases its affinity to ClpB.

Taken together, the results of the experiments in the presence of soluble and aggregated substrates indicate a strong coupling between substrate binding and M-domain dynamics. Remarkably, substrate binding to ClpB, which likely involves its previously characterized substrate binding sites on the N-terminal domain^77^ and at the central pore of NBD1^76^, exerts an allosteric effect that shifts the M domain toward the active conformation, enhancing the probability of DnaK binding to initiate the disaggregation activity. Nevertheless, this is done while the M domain continues to sample both active and inactive states, in fact at an increasing rate compared to the substrate-less state.

### Alteration of M-domain dynamics affects ClpB function

**Fig. 5:**
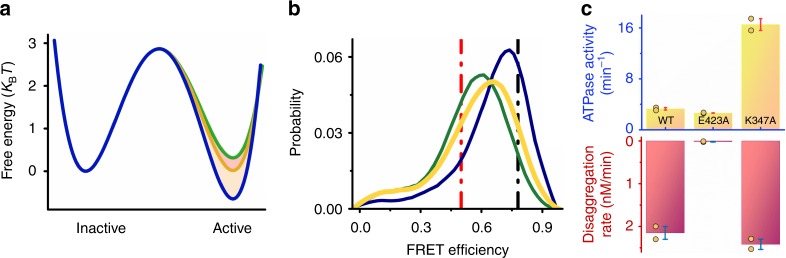
Mutations that change M-domain dynamics affect ClpB function. **a** Free-energy profiles of the M domain calculated from H^2^MM populations and rates as obtained from analysis of smFRET measurements of the WT (orange), K347A (blue), and E423A (green). In K347A state 1 is stabilized, while in E423A state 1 is destabilized (for all transition rates (in s^−1^) and their errors see Supplementary Table 4, for the population ratios see Supplementary Table 5). **b** FRET efficiency histograms of the WT (orange), K347A (blue), and E423A (green). The histogram of K347A is shifted to higher FRET efficiency values, while that of E423A is shifted to lower values. Vertical lines indicate the FRET efficiency values corresponding to state 1 and 2 from H^2^MM analysis of the WT measurement (Fig. 1E). **c** Steady state ATPase and disaggregation activity of the WT and the two mutants, demonstrating that changes in M-domain dynamics suppress disaggregation activity in E423A, and enhance ATPase activity in K347A. The disaggregation rate is expressed in terms of the concentration of G6PDH recovered per minute. Orange circles in (**c**) represent separate repeats of the measurements. Error bars represent standard errors of the mean. Source data are provided as a Source Data file

It is well-established that the M domain is important for the disaggregation function of ClpB. Indeed, it has been shown that crosslinks that hinder M-domain flexibility reduce disaggregation activity, though not ATPase activity^26,35^. Since we are now able to measure the dynamics of transitions of the M domain between active and inactive states, it is interesting to ask whether changes in dynamics are the cause of changes in the activity of ClpB. To answer this question, we turned to known ClpB mutants that activate or repress the protein. First, we generated the mutant K347A^29,81^. It was suggested that a salt bridge between residue K347 on NBD1 and a negatively charged residue (likely D471) on the M domain is broken in this mutant, leading to hyperactivation of ClpB^27,29,31,81^. smFRET measurements of the mutant protein showed that the transition rate from state 1 to state 2 decreased (*k*_12_ = 3100 ± 200 s^−1^), while the transition rate from state 2 to state 1 was almost similar to the WT (*k*_21_ = 5500 ± 200 s^−1^) (Fig. 5a, Supplementary Fig. 9H, Supplementary Table 4). This change leads to an increase of the population of the active state compared to the inactive state (Fig. 5b, Supplementary Table 5). Remarkably, this mutant was found to be hyperactive in terms of its ATPase activity (5 folds higher than the WT), though it showed similar disaggregation activity to the WT (Fig. 5c), as also reported by Hayashi et al.^82^.

We also mutated residue E423, which was suggested to be involved in a network of salt bridge interactions between the M domain and NBD1 of the same subunit, as well as between motif 1 of the M domain of one subunit and motif 2 of the M domain of an adjacent subunit^29,83^. H^2^MM analysis of smFRET results of E423A showed a significant increase in the transition rate from state 1 to state 2 (*k*_12_ = 7000 ± 350 s^−1^), leading to a decreased population of the active state (Fig. 5a, b, Supplementary Fig. 7H, I, Supplementary Fig. 9I, Supplementary Fig. 10D, H, L, Supplementary Table 2, and Supplementary Tables 4, 5). Indeed, this mutant possessed almost similar ATPase activity to the WT, but completely lacked disaggregation activity (Fig. 5c), likely due to deficient DnaK binding^30^ (the presence of DnaK did not change the FRET histogram of this mutant). The above studies with these two mutants and the two Walker A mutants (Fig. 3) demonstrated that changes in the relative population of the active state of the M domain affect the activity of ClpB, by modifying either its ATPase rate, or its disaggregation rate.

**Fig. 6:**
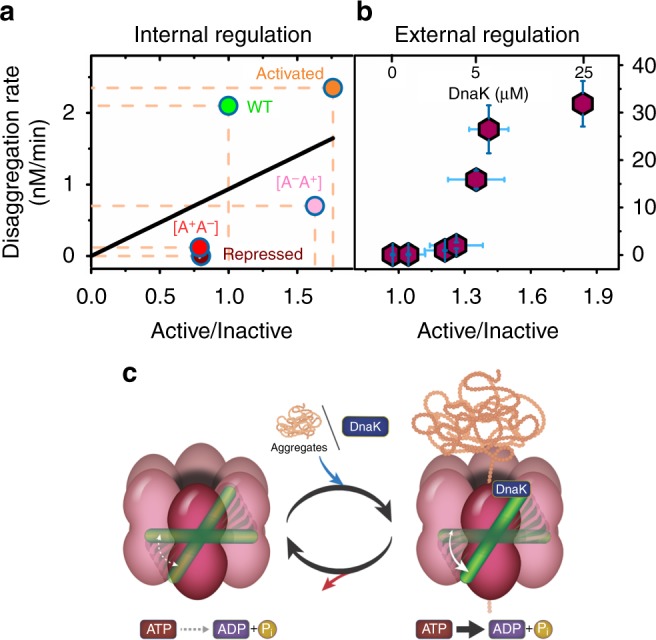
Tuning of the M-domain population ratio controls ClpB disaggregation rate. **a** Internal regulation: a map of the disaggregation rate vs. the active/inactive population ratio, obtained with different ClpB mutants, displays a high correlation coefficient of 0.94 ± 0.3, based on a linear fit to the data points (black line). **b** External regulation: disaggregation activity increases sigmoidally with the population ratio, here controlled by the concentration of DnaK. **c** Schematic of the paradigm for the operation of the M domain as a tunable switch that determines the level of activity of the machine. The M domain is in a dynamic equilibrium between the active and inactive states, rather than statically populating one of them. In the presence of ATP only (left) the ratio between the states is ~1, facilitating DnaK approach and binding. The addition of DnaK or aggregates (right) changes the ratio between the states by stabilizing the active state in a dose-response manner, which in turn tunes the machine’s activity level. Error bars represent standard errors of the mean

## Discussion

We studied here the dynamics of the M domain of the disaggregation machine ClpB. Using smFRET spectroscopy, we demonstrated that the M domain toggles between two major states on a very fast time scale of ~150 µs. This surprising finding suggests that some large-scale motions in proteins might be faster than previously thought. In fact, we recently reported even faster domain closure dynamics in the enzyme adenylate kinase^21^. In the current work, we used FRET efficiency values obtained from three separate donor-acceptor pairs to provide a structural model for the M domain in its two states, which could be identified with the active and inactive forms of the domain. This finding is particularly relevant for the structure of the active state, which was fully or partially missing in recent cryo-EM structures of ClpB. Most interestingly, we demonstrated extensive allosteric interactions between the NBDs of ClpB as well as its substrate binding sites and the M domain.

We found no evidence for the exclusive population of a single conformation of the M domain, as previously proposed^27–29,83,84^. Rather, both active and inactive states were populated, and they interchanged on a time scale that was much faster than any other activity of the chaperone, either ATP hydrolysis or disaggregation activity. These fast dynamics are important for the machine activity, because they allow DnaK to bind the active conformation of the M domain. Stabilizing the M domain in the inactive conformation by head to tail interactions of neighbors^31,83^, would reduce the probability for DnaK to bind. The relative populations of the two states were found to be determined by their interconversion rates, *k*_12_ and *k*_21_, which could be modulated by different perturbations, either internal (Fig. 3) or external (Fig. 4). Importantly, we found that modulation of the population ratio of M-domain states affects the activity of ClpB (Fig. 6a, b). In particular, mutations that modulate the active/inactive state ratio also modulate ATPase or disaggregation activity. Even more interestingly, alteration of ATPase activity by mutation at one of the ATP binding sites modulates the active/inactive ratio of the M domain, which in turn changes the disaggregation activity. While these are two cases in which the active/inactive ratio is modulated through internal changes in the machine (Fig. 6a), external control was also demonstrated. Indeed, increasing aggregate concentration up to 150 nM toggles the M-domain active/inactive ratio all the way to 1.85. Increased DnaK concentration also modulates the active/inactive ratio, thereby enhancing disaggregation activity (Fig. 6b).

These results suggest a paradigm for the operation of the M domain as a tunable switch that determines the level of activity of the machine (Fig. 6c). Rather than the static population of the active and inactive states of the M domain, a dynamic equilibrium between these two states is used as a signal to activate or repress the machine. The ultrafast dynamics of the M-domain, which are ~4–5 orders of magnitude faster than the machine activity, effectively turn it into a continuous switch, even though it continues to sample two discrete states. Further, a remarkable positive feedback mechanism is observed, by which ATP binding to NBD1 or NBD2 allosterically regulates M-domain dynamics (Fig. 3), while M-domain dynamics in turn regulate and enhance NBD activity (Fig. 5c). Future work will probe to what extent the dynamics of the M domains of different subunits of ClpB are coordinated, and how such coordination contributes to modulate machine activity.

The mechanism of tunable, continuous allosteric switching of ClpB’s M domain revealed here (which is similar to the mechanism suggested recently for a membrane protein^85^) involves relatively low free-energy barriers between the two states of the M domain, of the order of 3 K_B_T (smaller than the energy of a hydrogen bond, 6.7 K_B_T^86^, see Supplementary Table 11, but note that this estimate depends on the choice of the pre-exponent in Arrhenius equation). Such low free-energy barriers facilitate tuning of the states of the toggle, and therefore the activity of ClpB, by multiple effects, which may include aggregate formation and upregulation of DnaK expression during a heat shock response, as well as yet-unexplored factors such as changes in the level of cellular crowding. It is likely, though, that many other cellular machines^2,3,87–89^ adopt such a mechanism, whereby their activity is regulated by low free-energy barrier allosteric switches that are readily tuned by the cellular conditions.

## Methods

### Protein expression and purification

*Thermus thermophilus* ClpB (*TT* ClpB), DnaK, DnaJ, and GrpE DNA were cloned into a pET28b vector, with the addition of a six-histidine tag preceded by a tobacco etch virus protease (TEV) cleavage site. Common site-directed mutagenesis techniques were used to generate various mutants of ClpB^90^. See Supplementary Fig. 1A–D for protein characterization. For the sequence of ClpB with marked mutation positions see Supplementary Fig. 20. A complete list of the primers used is provided in Supplementary Table 12.

*E. Coli* BL21 (DE3) pLysS cells (from Invitrogen, catalog number C606003) were transformed with protein vectors and  grown to OD 0.9–1 at 37 °C. Expression was induced by adding 1 mM IPTG and the cells were then incubated at 25 °C overnight. Following expression, bacteria were harvested and the proteins were purified on a Ni-NTA resin (GE Healthcare) with an elution step involving 250 mM imidazole. This purification was followed by overnight dialysis in the presence of ATP to remove imidazole from the solution and assemble the protein. We further purified the protein using a HiPrep DEAE FF column equilibrated with 50 mM HEPES, 20 mM KCl, and 2 mM TCEP at pH 7.4 (DEAE buffer). The peak containing the purified protein was collected, and purity was assessed by gel electrophoresis (Supplementary Fig. 1A). From 1 L of growing bacteria we usually obtained 20–25 mg of pure protein.

We stored the protein until use at −80 °C at a concentration of 26 µM for ClpB, 650 µM DnaK, 40 µM DnaJ, and 125 µM GrpE. For experiments that required substrate and nucleotide-free ClpB and DnaK, we unfolded the protein at 6 M GdmCl to release any bound molecules, then refolded in steps using dialysis. Histidine-tag cleavage was done by adding 6 mg histidine-tagged TEV protease to 20 mg protein during the dialysis process. The cleaved protein was then separated from the protease and uncleaved protein on a Ni-NTA column.

### ClpB double-labeling process

We first labeled single-cysteine mutants of ClpB with Alexa 488 for 1 h and found that cysteines at positions 428 or 483 are less exposed to reaction with the dye compared to cysteines at positions 359 or 771. We could take advantage of this fact to semi-specifically label each site with the desired dye (donor or acceptor). 2 mg of ClpB in DEAE buffer were exchanged into the labeling buffer (25 mM HEPES, 25 mM KCl, pH 7.1) and then reacted with the acceptor dye molecules, Alexa 594 C5 maleimide, at a 1:1.2 protein to dye ratio for 1 h. ClpB molecules were then separated from the unreacted dye using a desalting column (Sephadex G25, GE Healthcare) equilibrated with the labeling buffer, with the addition of 2 M guanidinium chloride (GdmCl) to expose the partially buried sites for labeling. ClpB in 2 M GdmCl was then reacted with the donor dye Alexa 488 C5 maleimide at a 1:0.8 protein to dye ratio for 1 h. Unreacted dye molecules were then separated using a desalting column equilibrated with the labeling buffer including 2 M GdmCl.

To obtain ClpB hexamers with only a single labeled protomer or less, we reassembled ClpB molecules by mixing labeled subunits with cysteine-less subunits. The distribution of labeled vs. unlabeled protomers in mixed ClpB molecules was calculated based on the binomial distribution as follows^69^:

1 $$P\left( k \right) = \left( {\begin{array}{*{20}{c}} n \\ k \end{array}} \right)p^k(1 - p)^{n - k},$$

2 $$p = \frac{{ClpB_{{\rm{labeled}}}}}{{ClpB_{{\rm{labeled}}} + ClpB_{{\rm{WT}}}}},$$

where *P(k)* is the probability that each hexamer contains *k*-labeled protomers, *n* is the total number of protomers (*n* = 6), and *p* is the probability of protomer incorporation, defined as the ratio between the concentration of labeled and non-labeled protomers. To decrease the probability of incorporating two labeled protomers in each hexamer, we mixed the labeled ClpB with the non-labeled ClpB at a ratio of 1:100. Based on this ratio we expect the probability of incorporation of two labeled promoters in the same hexamer to be 0.15%, while the probability to find one labeled protomer in a hexamer is 5.7%.

To ensure a homogeneous mixing of these two groups of molecules, we mixed them in a 6 M GdmCl solution. The denaturant concentration was then reduced step-by-step using dialysis, until we reached 0 M GdmCl. Finally, the mixed subunits were further extensively dialyzed against the ClpB buffer to ensure their full refolding and reassembly. The 1:100 ratio of labeled to non-labeled ClpB molecules was verified by comparing the absorbance of Alexa 488 at 490 nm and the protein at 280 nm. It is important to mention that while in general we mixed double-labeled subunits with WT subunits, in the case of ClpB molecules that contained a functional mutation (K347A, E423A, [A^−^A^+^], [A^+^A^−^], and [B^−^B^−^], the double-labeled subunits were mixed with cysteine-less variants of the same mutant.

Native gel electrophoresis, gel filtration chromatography, and enzymatic activity assays showed that mixed labeled ClpB molecules were assembled and active to the same extent as the WT (Supplementary Figs. 1–3). The assembled molecules (at a total protomer concentration of 15 µM) were then filtered through 100 nm filters (Whatman Anotop-10), divided into 8 µl aliquots and stored at −80 °C.

### ATPase activity

To test the basal ATPase activity of ClpB, we determined the steady state ATP hydrolysis rate of the protein using a coupled colorimetric assay^91^. A volume of 1 µM ClpB (desalted to 50 mM HEPES, 50 mM KCl, pH 7.5) was incubated in the presence of various amounts of ATP (50 µM – 3 mM) in 50 mM HEPES (pH 7.8), 50 mM KCl, 0.01% TWEEN 20, and an ATP regeneration system (2.5 mM phosphoenol pyruvate, 10 unit/ml pyruvate kinase, 15 unit/ml lactate dehydrogenase, 2 mM 1,4 dithioerythritol, 2 mM EDTA, 0.25 mM NADH) at 25 °C. The reaction started by adding MgCl_2_ to a final concentration of 10 mM. ATP hydrolysis rate was then measured indirectly by monitoring NADH absorption at 340 nm over time. ATP hydrolysis rate was background subtracted and plotted as a function of ATP concentration and fitted to the Hill equation:

3 $$v = V_{{\rm{max}}}\frac{{S^n}}{{S^n + K_{0.5}^n}},$$

where *ν* is the initial reaction velocity, *V*_max_ is the maximum reaction velocity at saturating substrate concentration, *S* is the substrate concentration, *K*_*0.5*_ is the concentration at which half of the molecules are bound and *n* is Hill constant. The fit yielded a rate constant of 3.2 ± 0.10 min^−1^ and a Hill coefficient of ~3.1 ± 0.3 for WT ClpB, quite similar to literature values of 2.65 min^−1^ and 3.1, respectively^64^ (Supplementary Fig. 4A). The same ATPase activity assay was used for all studied mutants, which showed similar behavior to that of the WT, even when fully double labeled (Supplementary Fig. 4B).

### Disaggregation assay

This assay was adopted from ref. ^79^ with minor modifications: 90 µM G6PDH from *Leuconostoc mesenteroides* (Sigma Aldrich) was denatured in a buffer containing 50 mM HEPES, 5 M urea, 40 mM DTT, and 15% glycerol. G6PDH was then heated at 47 °C for 5 min. Aggregate formation was achieved by diluting (1:100) the denatured G6PDH into a reactivation buffer (50 mM HEPES, 30 mM KCl, 1 mM EDTA, 1 mM TCEP, 3 mM ATP, 20 µg/µl Pyruvate Kinase, and 20 mM MgCl_2_) followed by heating at 47 °C for 10 min^79^. We followed the aggregation formation step by light scattering using a spectrofluorometer (Supplementary Fig. 5A); results showed that aggregates indeed formed during the 10 min of incubation at 47 °C (Supplementary Fig. 5A). We also imaged these aggregates using transmission electron microscopy (Supplementary Fig. 5B), which showed very clearly amorphous aggregates.

To measure the disaggregation activity of our ClpB, we incubated 750 nM G6PDH aggregates with 2 µM ClpB, 2 µM DnaK, 1 µM DnaJ, and 1 µM GrpE in the same reactivation buffer as indicated previously. The disaggregation reaction was then initiated by incubating the reaction mix at 37 °C. The recovered activity of G6PDH was then measured at different time points as follows: 2 µl of the reaction mix was mixed with 198 µl of G6PDH activity assay (5 mM MgCl_2_, 2 mM D-glucose-6-phosphate and 1 mM NADP^+^). G6PDH activity was then monitored by following the formation of NADPH at 340 nm over time (10–30 min). Next, we divided the slope of each measurement by the slope of the native G6PDH activity at the same concentration to obtain the G6PDH recovery percentage, which was converted later to concentration. To obtain the disaggregation rate of ClpB, we plotted the concentration of the recovered G6PDH as a function of time. The disaggregation rate was then calculated from the initial linear part of the curve. Results showed that only when ClpB was present with all other co-chaperones was the disaggregation machinery active, yielding a disaggregation rate of 2.1 nM/min (Supplementary Fig. 5C). We also tested the disaggregation activity of our ClpB mutants. Results showed that all the mutants used in this study, including the fully double-labeled ClpB complex variants, were indeed active (Supplementary Fig. 5D).

### Sample preparation for smFRET experiments

Flow cells for single-molecule experiments were prepared from the assembly of two cover slip glasses of 24 × 50 mm and 18 × 18 mm. Both cover slips were rinsed with 10% hydrogen fluoride solution for 40 s and then washed with pure water. Dried cover slips separated by Teflon strips were glued together by 10 min incubation in a dry oven at 115 °C. Ready-to-use flow cells were washed three times with the smFRET buffer that contained 25 mM HEPES (pH 7.8), 10 mM MgCl_2_, 25 mM KCl, and 0.01% TWEEN. To avoid protein sticking to the surface we coated the flow cell surface with a supported lipid bilayer, by flowing in a solution containing liposomes prepared from egg phosphatidylcholine (Avanti Lipids) by extrusion through disposable 0.1 mm Anopore syringe filters (Whatman Anotop-10). The lipid solution was incubated within the cells for 4 min, after which the cells were washed three times with the smFRET buffer. Finally, we loaded 100 µl of ClpB at a labeled-subunit concentration of 50 pM, equivalent to an overall concentration of 5 nM. ClpB was diluted in the smFRET buffer with the addition of 2 mM ATP. We then sealed the flow cell with silicon grease to avoid dehydration^21,92^.

### Single-molecule FRET data acquisition and data analysis

Single-molecule measurements were conducted on freely diffusing molecules, using a home-built spectrometer^21,93^. In order to sample both donor and acceptor dyes, we used pulsed interleaved excitation with 485 nm and 594 nm diode lasers pulsed at a ratio of 3:1 and a repetition rate of 40 MHz. The emitted photons were divided into two channels according to their wavelengths using a dichroic mirror (FF580-FDi01; Semrock) and filtered by band-pass filters (ET-535/70 m for the donor channel and ET-645/75 m for the acceptor channel, both from Chroma). Arrival times of these photons were registered by two single-photon avalanche photo-diodes (Perkin Elmer SPCM-AQR-15) coupled to a standalone time-correlated single-photon counting module (HydraHarp 400, PicoQuant).

We detected fluorescent bursts in the single-molecule data using methods developed in the lab^21,94^. Briefly, the task of separating background photons from those related to fluorescent bursts was facilitated by first smoothing the data with a running-average of 15 photons. A cut-off time of 10 µs was determined from the histogram of the time lags, and used to effectively find the start and end points of each burst. Only fluorescence bursts with a total of 30 photons or more were selected for further analysis. Data were not corrected for channel crosstalk or background. From each measurement we obtained 12,000–16,000 burst events, and the average photon flux calculated from all events was around 190,000 photons per second. The raw FRET efficiency of each burst was then calculated based on the photons detected in both channels after donor excitation only, and the raw stoichiometry was calculated from the detected photons in both channels after both excitations, as described elsewhere^95,96^. A 2D histogram of raw stoichiometry versus raw FRET efficiency was generated (see 2D histograms of different ClpB mutants and conditions in Supplementary Fig. 6), from which we calculated the amount of emitted donor photons leaking into the acceptor channel, and the level of direct excitation of the acceptor dye by the 485 nm laser excitation^96^. We corrected the photon stream in both channels based on the calculated correction factors. To obtain a corrected FRET histogram without the donor and acceptor only populations, we selected only photon bursts with a stoichiometry that corresponded to molecules with both donor and acceptor dyes (Supplementary Fig. 6).

To extract the dynamics hidden in the FRET efficiency histograms, we used the H^2^MM algorithm as described in refs. ^21,42^ (the computer code for H^2^MM Matlab files can be found in https://pubs.acs.org/doi/abs/10.1021/acs.jpcb.6b10726). For this analysis, we took the filtered double-labeled molecules, and used photons arising from donor excitation only. Line plots of experimental FRET efficiency histograms and recoloring histograms, were smoothed using Savitzky–Golay filtering with a 12-point window (see Supplementary Fig. 9 for unsmoothed versions of all line plots).

### M-domain structural model based on smFRET measurements

We developed an analysis scheme that allowed us to obtain the structure of the M domain in its various states from smFRET measurements. Using the structural model of *TT*. ClpB^54^ and assuming rigid body motions of the M domain, we created a large number of potential M domain conformations by rotating one of the M domains around its anchoring point to NBD1 (residue 396). A set of 15,625 conformations was initially generated, and we then excluded all conformations that caused a steric clash within the same protomer or between adjacent protomers. A clash was defined as a van der Waals (VDW) contact between M domain atoms and other atoms of the ClpB hexameric complex.

4 $$0 \le d_{ij} \,< \,R_v\left( i \right) + R_v\left( j \right) - 0.3,$$

where *R*_*v*_(*i*) and *R*_*v*_(*j*) are the VDW radii of atoms *i* and *j*, respectively, and *d*_*ij*_ is the distance between the two atoms. The factor 0.3 Å is our clash threshold value, which was calculated from the raw structure of the hexameric model^53^.

Following the procedure above, the conformational space was reduced to only ~5% of all the 15,625 conformations. We then calculated the FRET efficiency values for the three FRET pair locations (S428C-S771C, S359C-S428C, and S359C-Q483C) in each of the allowed conformations.

To optimize our model further, we took the dye and linker orientation into consideration. This was done using the accessible volume (AV) method developed by Michaelis and coworkers^49,50^. We calculated the AV for each dye to get a cloud of allowed position in each conformation (see Supplementary Notes for more details). We then calculated the FRET efficiency value for each pair from all possible dye positions (600–1200 positions in each cloud) using a Förster distance (*R*_0_) separately calculated for each FRET pair (based on data measured experimentally from same labeled ClpB molecules, see Supplementary Table 13 for then values for each FRET pair and other photophysical properties of the dyes). Finally, we calculated the average FRET efficiency value for each FRET pair based on all these positions, and the chi-squared value defined as follows:

5 $$\chi _E^2 = \mathop {\sum}\nolimits_{i = 1}^n {\left( {E_{{\rm{measured}}}(i) - E_{{\rm{model}}}(i)} \right)^2} ,$$

where *n* = 3 is total number of FRET pairs, *E*_measured_(*i*) is the measured FRET efficiency value obtained from the H^2^MM analysis for FRET pair *i*, and *E*_model_(*i*) is the calculated FRET efficiency value for the same pair. The chi-squared values were calculated twice, once with *E*_measured_(*i*) values of state 1 and the second time with the *E*_measured_(*i*) values of state 2 (Supplementary Table 3 and Fig. 2b, c).

### Reporting Summary

Further information on experimental design is available in the Nature Research Reporting Summary linked to this article.

## Supplementary information

Supplementary Information

Reporting Summary

Source data file

## Data Availability

Data supporting the findings of this manuscript are available from the corresponding author upon reasonable request. A reporting summary for this Article is available as a Supplementary Information file. The source data underlying Figs 1e, 2b–c, 3a–c, 4a–f, 5a–c, 6, and Supplementary Fig. 8, Supplementary Tables 3–10 are provided as a Source Data file.
