# Supplementary material for: Tunable microsecond dynamics of an allosteric switch regulate the activity of a AAA+ disaggregation machine
Source: Nat Commun. 2019 Mar 29;10:1438. doi: 10.1038/s41467-019-09474-6 (PMC6440998; doi:10.1038/s41467-019-09474-6)
Supplement: Supplementary file 1 — Supplementary Information [file 41467_2019_9474_MOESM1_ESM.docx]

Tunable Microsecond Dynamics of an Allosteric Switch Regulate the Activity of a AAA+ Disaggregation Machine

Hisham Mazal^1^, Marija Iljina^1^, Yoav Barak^2^, Nadav Elad^2^, Rina Rosenzweig^3^, Pierre Goloubinoff^4^, Inbal Riven^1^ and Gilad Haran^1*^

^1^Department of Chemical and Biological Physics, Weizmann Institute of Science, Rehovot 761001, Israel. ^2^Department of Chemical Research Support, Weizmann Institute of Science, Rehovot 761001, Israel. ^3^Department of Structural Biology, Weizmann Institute of Science, Rehovot 761001, Israel. ^4^Department of Plant Molecular Biology, Faculty of Biology and Medicine, University of Lausanne, CH-1015 Lausanne, Switzerland.

* Corresponding author: gilad.haran@weizmann.ac.il

Supplementary Information

### **Supplementary Notes**

### **ClpB assembly tests.** ClpB assembly, described in the Methods section, was confirmed by several methods. First, we ran the assembled, double-labeled ClpB on a native gel composed of 3% acrylamide at 35 V and 4 °C, in the presence of 10 mM MgCl_2_ and 2 mM ATP. Only a single band was observed (Supplementary Figure 1 D-F), indicating that the protein assembly was homogeneous for all ClpB mutants and double-labeled variants.

Second, we ran the double-labeled ClpB S428C- S771C mixed 1:100 with WT ClpB on a gel filtration Superdex 200 10/300 GL column (GE Healthcare) equilibrated with 25 mM HEPES, 25 mM KCl 10 mM MgCl_2_ and 2 mM ATP (ClpB buffer). The elution profile of ClpB was measured by absorbance at 280 nm, 488 nm and 594 nm using Äkta Pure Chromotography system (GE Healthcare). The oligomerization state of ClpB (580 kDa for hexamer assembly) was roughly estimated based on the elution volume of the reference protein thyroglobulin (669 kDa). ClpB eluted as a single peak at 9.75 ml, which was very close to the elution volume of thyroglobulin (9.45 ml), indicating that the protein was assembled. The elution profile of the double-labeled ClpB was found to be similar to the WT (Supplementary Figure 1G).

We also analyzed ClpB oligomerization by using transmission electron microscopy of negatively stained particles. A 3.5 µl sample of ClpB at a concentration 200 nM in the presence of 2 mM ATP was applied to glow discharged, carbon coated, 300 mesh copper grids, followed by a 30-second incubation. Excess liquid was blotted off and the grids were stained with 2% uranyl acetate. Imaging was done with FEI Tecnai T12 electron microscope using a Gatan OneView Camera (Supplementary Figure 2A). For cryo-EM imaging 3.5 μL of a 200 nM ClpB solution were applied to glow-discharged Quantifoil holey carbon grids (R2/2, 200 mesh) coated with a thin layer (~2 nm) of carbon, and plunge frozen in liquid ethane cooled by liquid nitrogen, using a Leica EM-GP plunger (3.5 s blotting time, 80% humidity). Grids were imaged at liquid nitrogen temperature on an FEI Tecnai TF20 electron microscope operated at 200 kV with a Gatan side entry 626 cryo-holder. Dose-fractionated movies were recorded on a K2 Summit direct detector (Gatan) mounted at the end of a GIF Quantum energy filter (Gatan) using a slit of 20 eV. 15 Movies were recorded using a low dose procedure implemented in SerialEM ^1^, frames were aligned (5×5 tiles) and summed using MotionCor2 ^2^, followed by contrast transfer function (CTF) estimation using CTFFIND4 ^3^. Class averages from 6592 particles were generated using RELION 2.0 ^4^ (Supplementary Figure 2B).

**Validating the structural integrity of ClpB hexamers.** The success of the experiments with reassembled ClpB molecules depends on the lack of exchange of subunits between molecules and stable assemblies. Some authors reported facile subunit exchange in ClpB ^5-7^, and others reported assembly destabilization of ClpB Walker A mutants ^8-10^. These results are likely due to the conditions used, such as high salt concentrations (NaCl/KCl above 150 mM). Therefore, we tested for disassembly and mixing of subunits between ClpB hexamers under the exact conditions of our experiments, using low salt concentration (25 mM HEPES, 25 mM KCl, 10 mM MgCl_2_ and pH 8). A single-cysteine mutant (S771C) was purified and assembled. Then, we labeled one portion of the protein with Alexa 488 C5 maleimide (Invitrogen) at a ratio that guaranteed that essentially all subunits would be labeled, and a second portion with Alexa 594 C5 maleimide (Invitrogen) in a similar manner. Based on the published model for the ClpB hexamer, the distance between adjacent labeled sites (which are on adjacent protomers) is 56.53 Å (Supplementary Figure 3A). Thus, if there were subunit exchange, we would expect to observe some energy transfer following mixing of the two types of labeled molecules. We mixed these pre-assembled molecules in a 2 mM ATP solution at a 1:1 ratio and a total concentration of 5 nM (similar to the total concentration in the single-molecule experiments) for 24 hr at 25 °C. We then conducted smFRET measurements at a 50 pM concentration. The results showed a single peak at a FRET efficiency value of 0 (Supplementary Figure 3B, green line), indicating that no mixing occurred under these conditions, similar to what was recently proposed in the literature ^5^. As a positive control for subunit mixing, we mixed ClpB molecules labeled with the two dyes as above in a 6 M GdmCl solution. The molecules were then refolded and assembled in the presence of 2 mM ATP. smFRET measurements showed a broad peak at intermediate FRET efficiency values, which indicated mixed complexes (Supplementary Figure 3B, red line). We conducted the same experiment as described above (without denaturant), but without ATP. Interestingly, a broad FRET signal was observed here as well (Supplementary Figure 3B, blue line), an indication of disassembly and subunit mixing in the absence of nucleotides.

Next, we tested also whether DnaK binding can destabilize ClpB complexes and cause subunit mixing under the conditions of our experiments. We mixed and incubated pre-assembled ClpB molecules (labeled as above with donor and acceptor dyes) at a total concentration of 5 nM in the presence of 2 mM ATP and 7 µM DnaK for 24 hrs. The smFRET measurement of this mixture showed no indication of subunit mixing (Supplementary Figure 3B, orange line).We repeated the same experiments for Walker A mutant [A^-^A^+^] (Supplementary Figure 3C - violet line- with ATP, green line- without ATP), and Walker A mutant [A^+^A^-^] (Supplementary Figure 3D - pink line- with ATP, brown line- without ATP). The results showed again that these ClpB molecules were properly assembled and did not mix in the presence of ATP.

As another way to test whether our measurements were affected by the low concentration of ClpB molecules, we conducted smFRET experiments on the labeled S428C-S771C construct in the presence of increasing concentrations of non-labeled ClpB WT molecules (0.2 and 1 µM). Only a minor change in the shape of the FRET histograms was registered, an indication of proper assembly (Supplementary Figure 3E). We repeated the same experiment for ClpB Walker A mutants, and as expected no changes of the FRET histograms were registered in the presence of increasing concentrations on non-labeled ClpB molecules (Supplementary Figure 3 F-G). Finally, in the absence of ATP the FRET histogram of the S428C-S771C labeled construct changed dramatically (red line in Supplementary Figure 3H).

Taken together, these results indicated that under native conditions and in the presence of ATP, ClpB remains well-assembled even at a concentration of 5 nM.

**ClpB ATPase activity stimulation.** We tested the stimulation of ATP hydrolysis of ClpB in the presence of the protein substrate κ-casein (purchased from Sigma Aldrich)^11,^ ^12^. ClpB (1 µM) was incubated with the ATP regenerative system (see Methods in main text) in the presence of [0.5 – 230 µM] κ-casein. Indeed, ClpB exhibited an increase in the basal ATPase activity, in the presence of increasing concentration of κ-casein (Supplementary Figure 4C).We also tested the ATPase activity of ClpB near its optimum temperature (40 °C)^13^. Results showed that the ATP hydrolysis rate is stimulated 10 folds relative to the rate at 25 °C (Supplementary Figure 4D). The same ATPase activity assay was used for all the other studied mutants, which showed similar behavior to that of the WT ClpB.

**Dye linker simulations.** We modeled the dye positions using the method developed by Michaelis and coworkers and described in refs. 14,15 of the articles. In particular, we calculated the accessible volume of the dye-linker combination attached to the sulfur atom of cysteine residues following ‘mutation’ using Pymol (for the mutants S428C, Q483C, S359C and S771C). We used dye parameters for Alexa 488 C5 maleimide and Alexa 594 C5 maleimide provided in ref. 16: linker length of 20.5 Å and diameter of 4.5 Å, dye diameter of 8.5 Å. The accessible volumes were modeled on a lattice spanning -150 to 150 Å in each dimension with a spatial resolution of 2 Å. In the end of the calculation, we obtained a cloud of coordinates, which demonstrate the possible dye locations.

**Testing the suitability of the three-state model.** In addition to the various validation methods mentioned in the main text, we set to test that the main dynamics involving the discrete states 1 and 2 could not actually be described by a single state involving diffusion in a harmonic potential well. To this end, we generated simulated data by recoloring photon trajectories assuming such a model, but with the same photon arrival time of our real data. The simulation was performed using a simple Metropolis Monte-Carlo approach. In particular, we modeled the diffusion as the damped motion of a spring with a spring constant of 0.001 *K_B_T Ǻ*^-2^, and a relaxation time of 150 µs. With these parameters and an appropriate choice for the equilibrium distance, we could obtain a FRET histogram with a similar width to the major peak of the experimental histogram (Supplementary Figure 21 A). We analyzed the simulated data using H^2^MM with a two state model and then performed segmentation analysis. Indeed, this analysis showed two separate peaks. This might not be that surprising, as motion in a single potential can still be artificially segmented into two ‘states’ (this is in contrast to a true single-state FRET peak, where we have shown time and again that the analysis finds only one state). Interestingly, though, we found that the analysis with a two-state model was not stable: when we increased the number of states in the model, the positions of the states shifted (Supplementary Figure 21 B-C). In contrast, when we analyzed the experimental data in the same way, the solution obtained seemed more stable, and the states did not shift (Supplementary Figure 21 D). This result strengthened our confidence that a model with two discrete states better describes our data.

### **Supplementary Figures**


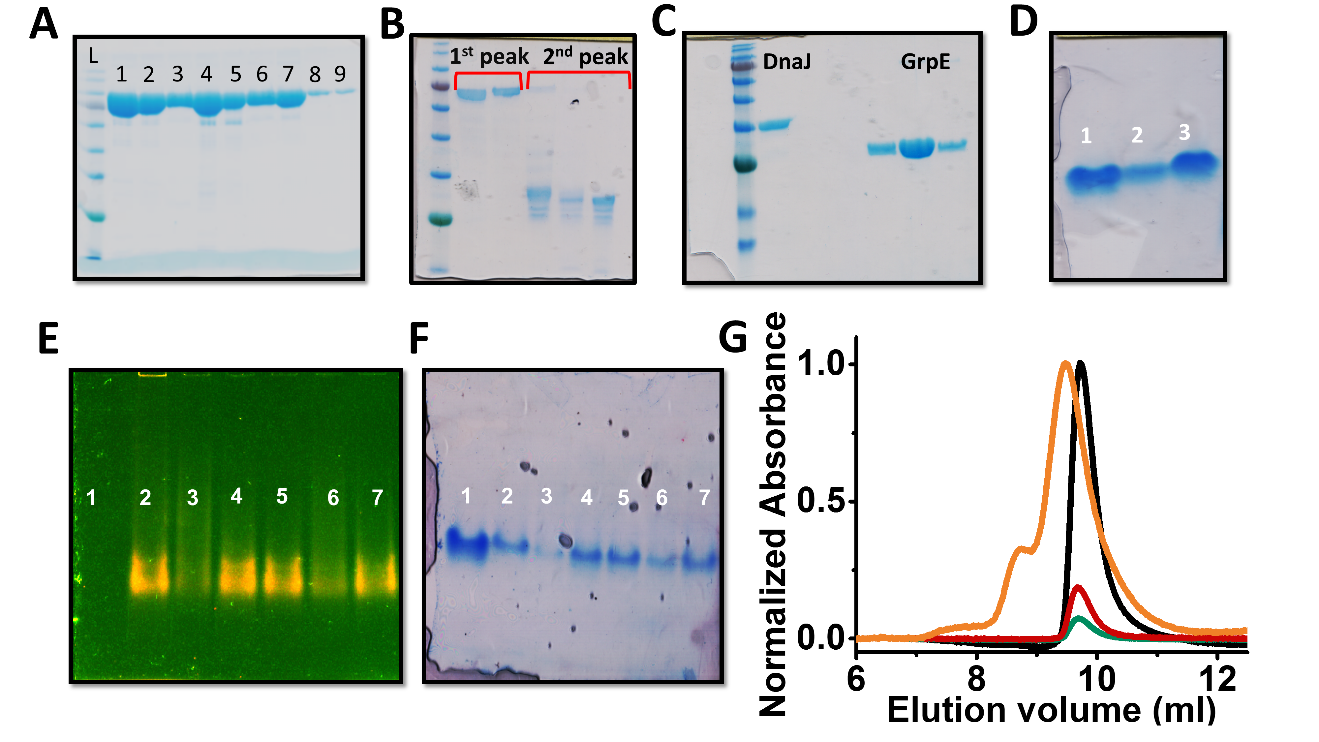


**Supplementary Figure 1. Purification and characterization of ClpB and co-chaperones.** (A) SDS gel electrophoresis of fractions of *TT.* ClpB after separation on a DEAE column. The left lane (L) is the PM2500 (SMOBIO) ladder, and the other lanes are different mutants of ClpB. 1 – S428C-S771C, 2 – S428C-S359C, 3 – Q483C-S359C, 4 – K347A, 5 – E423A, 6 – [A^-^A^+^], 7 – [A^+^A^-^], 8 and 9 are same as 1 and 2 but with a lower protein concentration. (B) SDS gel of DnaK after separation on DEAE column. The left lane is the ladder PM2500, the lanes labeled “1^st^ peak” are from the eluted first peak, which contained pure DnaK with the correct molecular weight, and the lanes labeled “2^nd^ peak” are from the second eluted peak, which contained contamination proteins bound to DnaK. (C) SDS gel of DnaJ and GrpE after final separation on DEAE column. The three GrpE lanes are different fractions from the same eluted peak. (D) Native gel electrophoresis (3 % acrylamide), ran in the presence of 10 mM MgCl_2_ and 2 mM ATP. WT *TT.* ClpB (1), a single cysteine mutant (S771C, 2) and a double cysteine mutant (S428C–S771C, 3). Each lane in the gel shows only one band indicating a homogenous solution of the assembled proteins. (E-F) Native gel (3 % acrylamide) of double-labeled ClpB (S428C–S771C), ran under the same conditions as in D. The gel was imaged with a Typhoon scanner at 473 nm and 532 nm (E). The combination of the two scans presented a yellow band indicating a double-labeled complex. The gel was then stained with Coomassie Blue (F). Lane 1 is WT ClpB, which we could observe with the Coomasie stain but not with fluorescence, 2-3 are different concentrations of the double labeled ClpB S428C-S771C doped in 1:100 WT ClpB, 4 is double labeled ClpB S359C-Q483C mixed 1:100 with WT ClpB, 5 is double labeled ClpB S359C-S428C mixed 1:100 with WT ClpB, 6 is double labeled ClpB (S428C-S771C) inactive mutant E423A mixed 1:100 with non-labeled inactive mutant and 7 is double labeled (S428C-S771C) ClpB hyperactive mutant (K347A) mixed 1:100 with non-labeled hyperactive mutant. We only observed one band in each case, indicating homogeneity of the samples. (G) The oligomerization state of double-labeled ClpB S428C- S771C mixed 1:100 with WT ClpB in the presence of 1 mM ATP. Thyroglobulin, with a molecular weight of 669 kDa served as a reference (orange). Absorption at 280 nm- black, 488 nm- green, 594 nm- red.


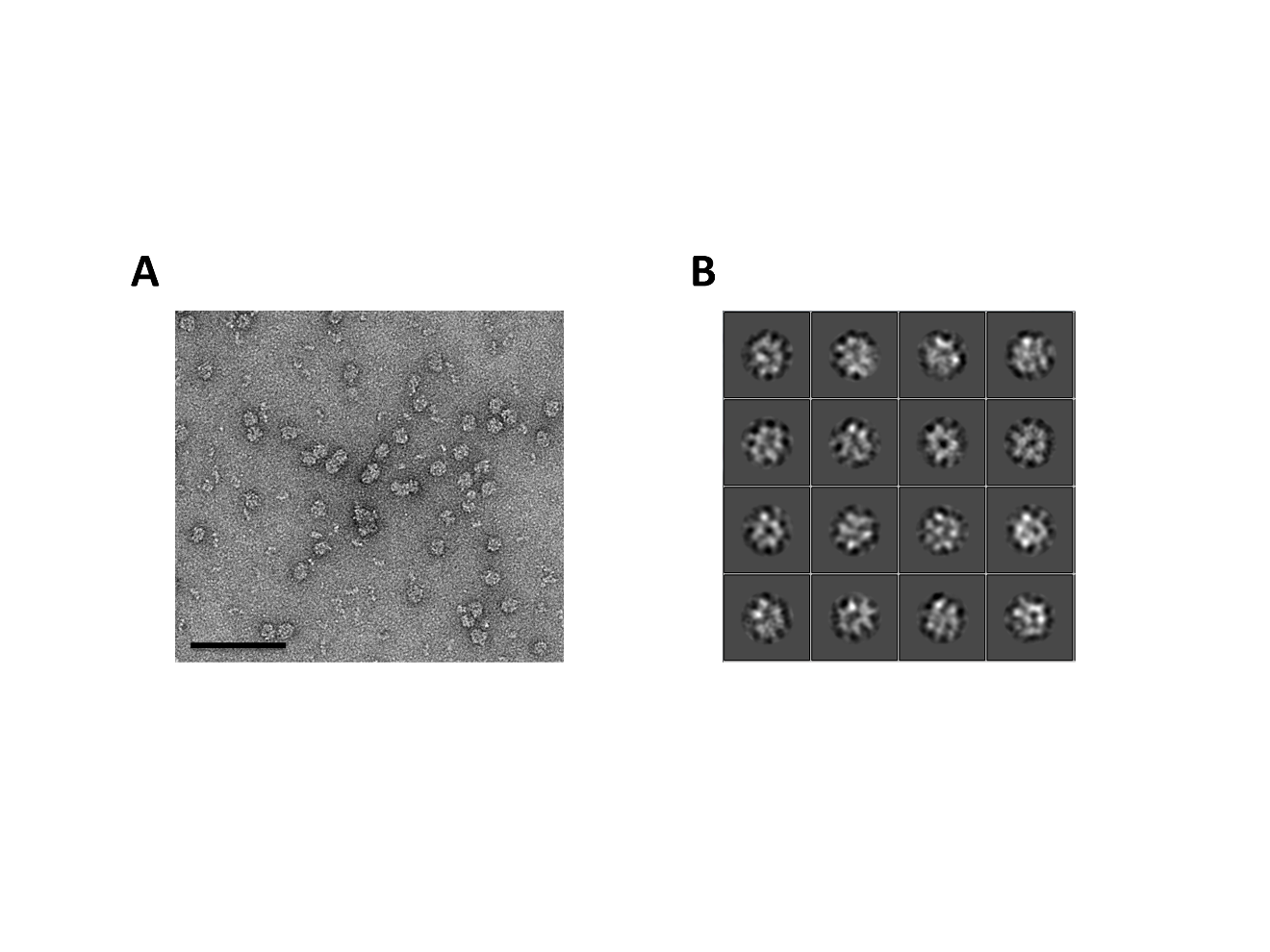


**Supplementary Figure 2. Analysis of ClpB assembly using electron microscopy (EM)**. (A) ClpB assembly characterization using EM of negatively stained samples. ClpB was imaged in the presence of 2 mM ATP, the scale bar is 100 nm. (B) ClpB assembly characterization using cryo-EM Representative 2D class averages generated from the data set showing multiple side and tilted views of assembled ClpB.


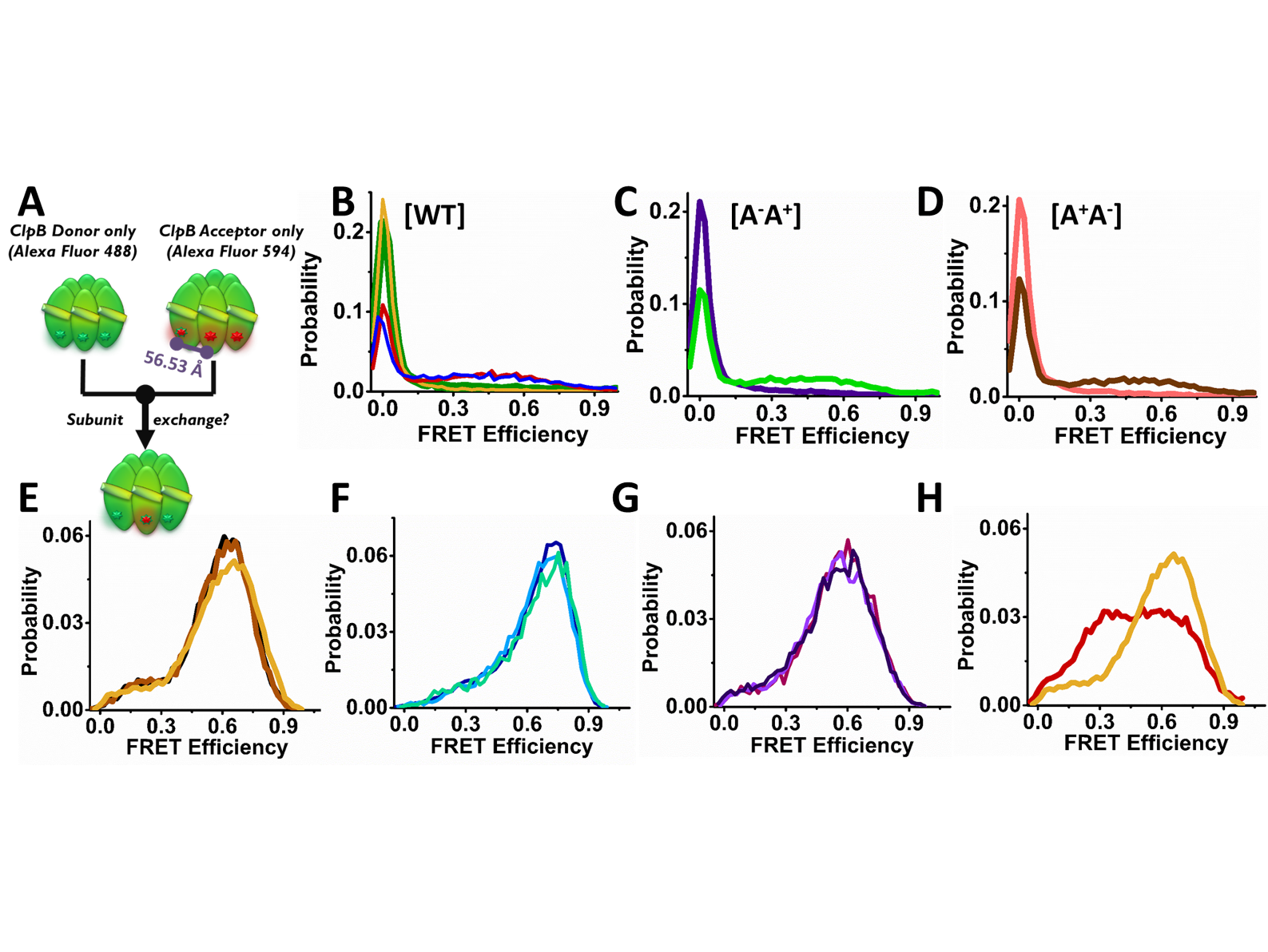


**Supplementary Figure 3. Validation of ClpB integrity under the smFRET experimental conditions.** (A) ClpB single cysteine mutant (S771C) was purified, assembled and labeled with Alexa Fluor 488 (Green) or Alexa Fluor 594 (Red), separately. (B-D) We then tested whether there was subunit exchange between the two complexes, incubating them together at a total concentration of 5 nM for 24 h at 25 °C, then measuring them using smFRET experiments. (B) Green line- in the presence of 2 mM ATP, blue line- no ATP, red line- positive control performed by incubation in the presence of 6 M GdmCl followed by refolding in the presence of 2 mM ATP, orange line- in the presence of 2 mM ATP and 7 µM DnaK. (C) Similar experiments with [A^-^A^+^], violet line- with ATP, green line- without ATP. (D) Experiments with [A^+^A^-^], pink line- with ATP, brown line- without ATP. (E-G) Experiments in the presence of increasing concentrations of non-labeled ClpB. (E) Orange line- no unlabeled protein added, brown line- 200 nM and black line- 1 µM. (F) Same experiments with [A^-^A^+^]. Dark blue line- no unlabeled protein added, light blue line – 200 nM, turquoise line - 1 µM. (G) Same experiments with [A^+^A^-^]. Violet line – no unlabeled protein added, bright-purple line - 200 nM and dark-purple line - 1 µM. (H) FRET efficiency histograms of ClpB in the absence of ATP (red line) compared to the histogram with ATP (orange line). The FRET efficiency histograms of the [A^-^A^+^] and [A^+^A^-^] mutants are clearly different from the histogram of ClpB in the absence of ATP, further supporting our finding that these mutants are well-assembled.


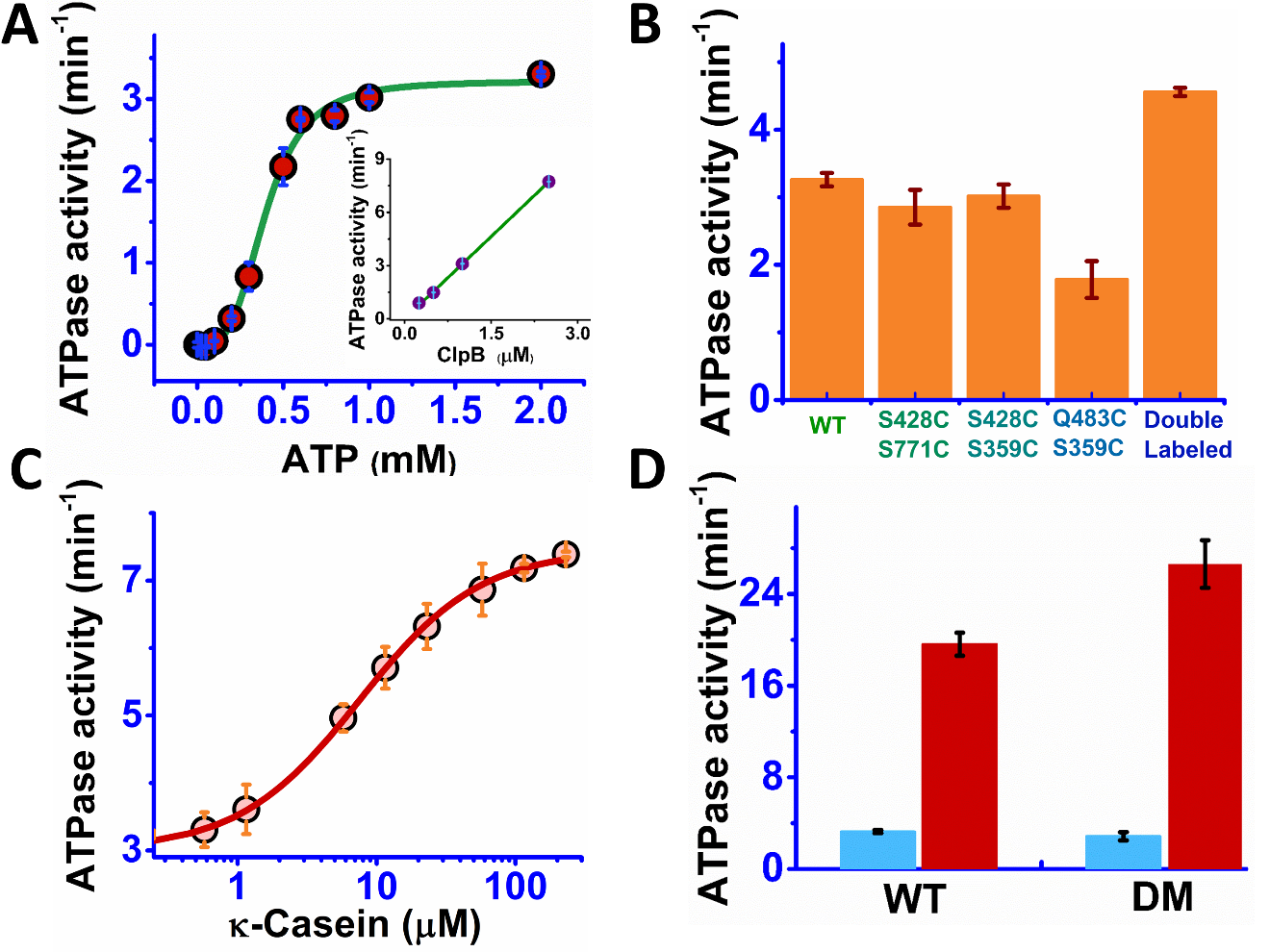


**Supplementary Figure 4. *TT.* ClpB ATPase activity.** (A) ClpB was incubated in the presence of various concentrations of ATP at 25 °C, and the initial ATP hydrolysis rate was measured and plotted. A fit to the Hill equation (equation 3 in Methods section, main text) yielded a rate constant of 3.2 ± 0.1 min^-1^, a K_0.5_ value of 390 ± 15 and a Hill coefficient of 3.1 ± 0.3, quite similar to literature values^17^. Inset is the ATP hydrolysis rate at 1 mM ATP as a function of ClpB concentration. The trend is linear as expected for an assembled protein. (B) ATPase activity of WT ClpB and the different double-cysteine mutants used in this work in the presence of 2 mM ATP and at 25°C. The last column (Double Labeled) is the fully double-labeled S428C-S771C mutant ClpB complex. All mutants have similar activity to the WT The error bars were obtained from two repeats of each experiment. (C) ClpB ATPase activity stimulation in the presence of κ-casein. ClpB was incubated at 25 °C in a similar buffer to our smFRET buffer, which contained 25 mM HEPES, 25 mM KCl, 10 mM MgCl2 as well as 2 mM ATP and the substrate κ-casein (0.5 - 230 µM). The presence of the substrate enhanced ClpB ATPase activity as expected for assembled and active ClpB. We fitted the measurements to a binding model, and obtained a K_d_ of 7.4 ± 0.2 µM, which represents the affinity of κ-casein to ClpB. (D) Increase in temperature (40 °C) stimulated WT ClpB ATPase activity 10 folds, as the temperature became closer to the physiological range (~55 °C). The non-labeled double cysteine mutant (DM, S428C-S771C) behaved in a similar manner to the WT. Blue- 25 °C, red- 40 °C. Errors shown here and in all the other figures represent standard errors of the mean.

**
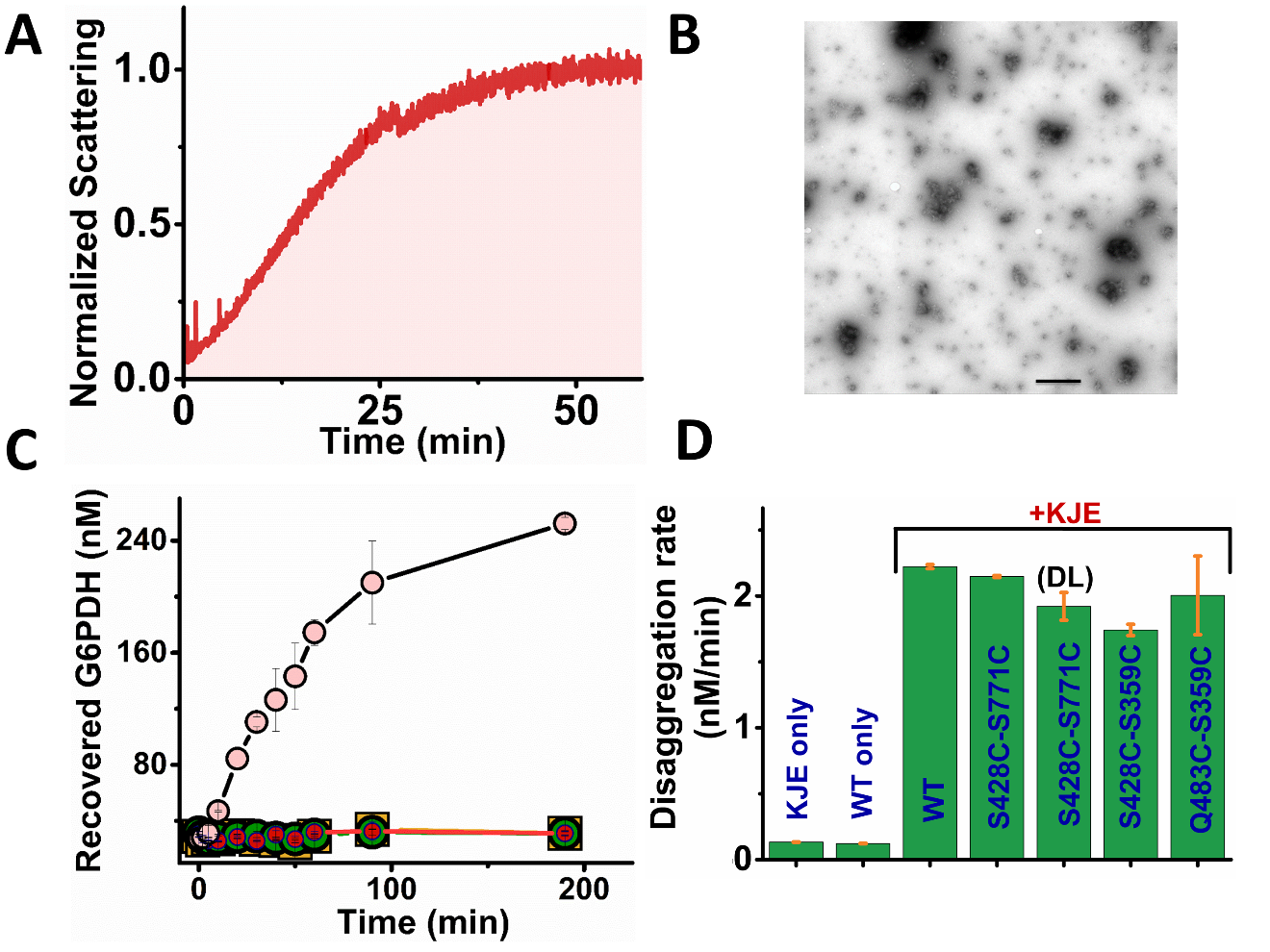
**

**Supplementary Figure 5. Characterization of G6PDH aggregates and disaggregation.** G6PDH aggregates were prepared by incubating 900 nM of unfolded G6PDH at 47°C. (A-B) Aggregate formation was monitored using two methods. (A) Light scattering was measured within a spectrofluorometer, with excitation and emission both set to 550 nm. The light scattering increased over time, an indication of aggregate formation. (B) G6PDH aggregates were imaged after 10 min incubation at 47°C, using negative-stain electron microscopy. The images clearly show heterogeneous aggregates of G6PDH. The scale bar is 500 nm. (C) Time course of G6PDH disaggregation activity. 750 nM G6PDH aggregates in a reactivation buffer (see Methods), were incubated with 2 µM ClpB, 2 µM DnaK, 1 µM DnaJ and 1 µM GrpE (KJE) for up to 3 hrs at 37°C, and enzymatic activity was measured at different time points. Pink circles- reactivation in the presence of ClpB + KJE, red circles- aggregates only, green circles- KJE only, orange rectangles- ClpB only. All control results showed only basal activity. (D) Disaggregation activity of ClpB mutants was measured with the procedure described in C. All mutants, including the fully double-labeled mutant ClpB complex (DL), were active in a similar way to WT ClpB.


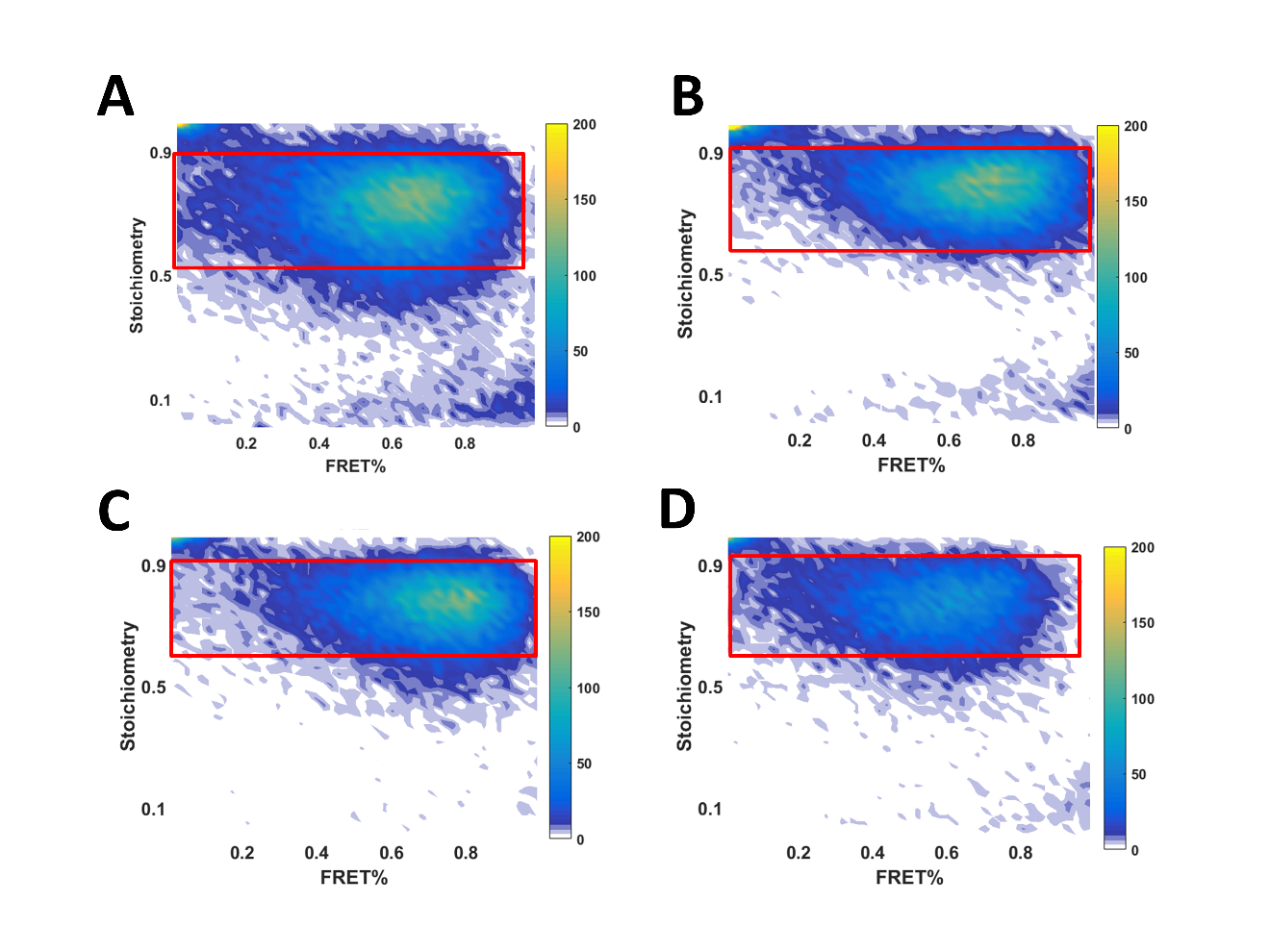


**Supplementary Figure 6. 2D FRET-stoichiometry histograms of ClpB molecules.** smFRET Experiments on different double-labeled ClpB mutants were conducted under various conditions and in the presence of 2 mM ATP. From each experimental data set, a 2D histogram of stoichiometry vs. FRET efficiency was created from ~12000-16000 burst events as described in Methods section. The double-labeled species is found at a stoichiometry of 0.7, the donor-only species at a stoichiometry above 0.96 and the acceptor-only species at a stoichiometry below 0.3. Only bursts of the double-labeled species were taken for further analysis (red rectangle). (A) ClpB (S428C-S771C). (B) ClpB (S428C-S771C) in the presence of 7 µM DnaK. (C) Active mutant ClpB (K347A, S428C-S771C). (D) Inactive mutant ClpB (E423A, S428C-S771C).


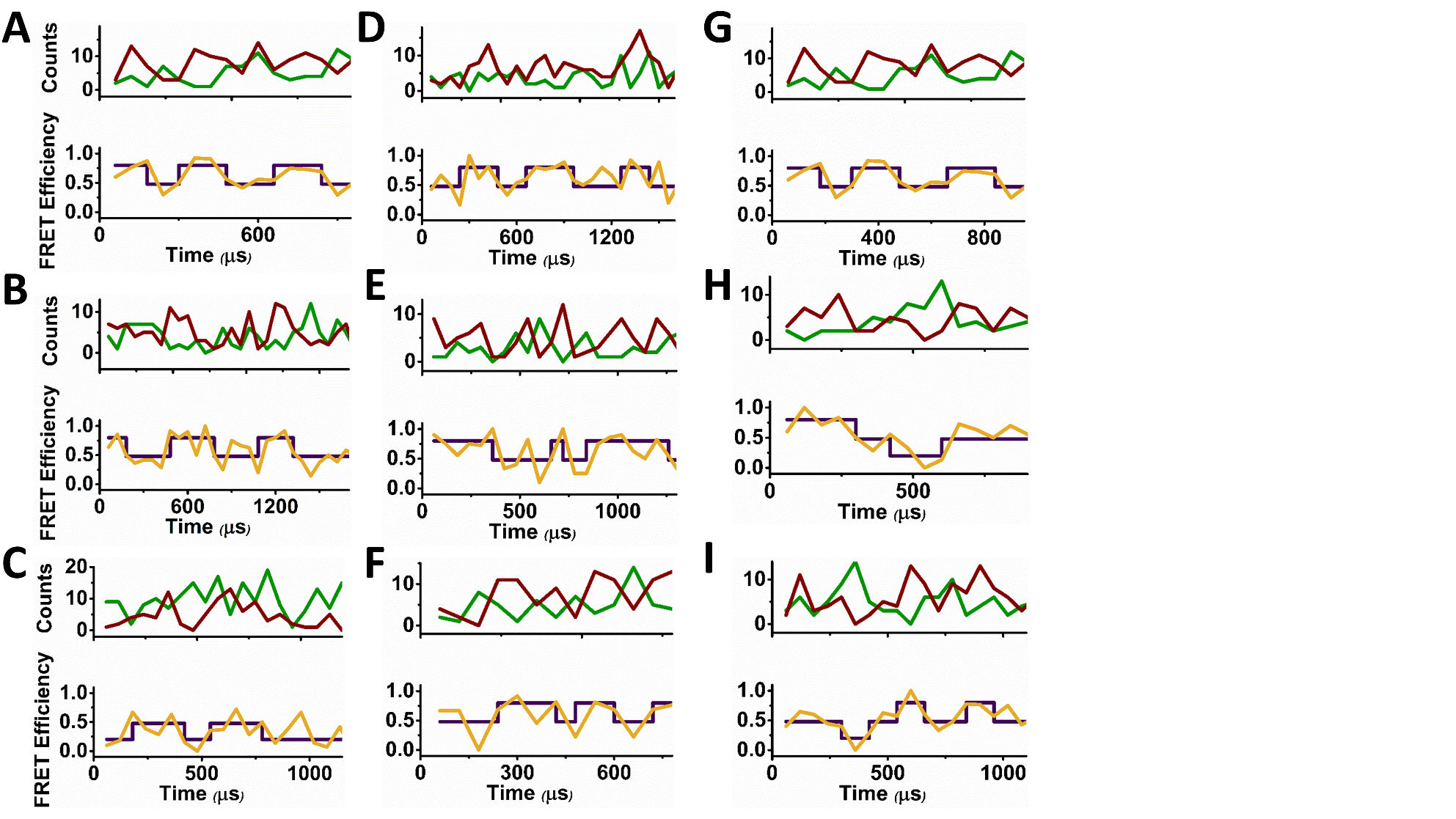


**Supplementary Figure 7. Single molecule trajectories.** Trajectories binned at 60 µs showing transitions between the three states. Top panel in each section shows the donor and acceptor signals (green and red, respectively), while the bottom panel shows the FRET efficiency obtained from the signals in orange, with the purple line representing state assignment obtained from H^2^MM analysis.


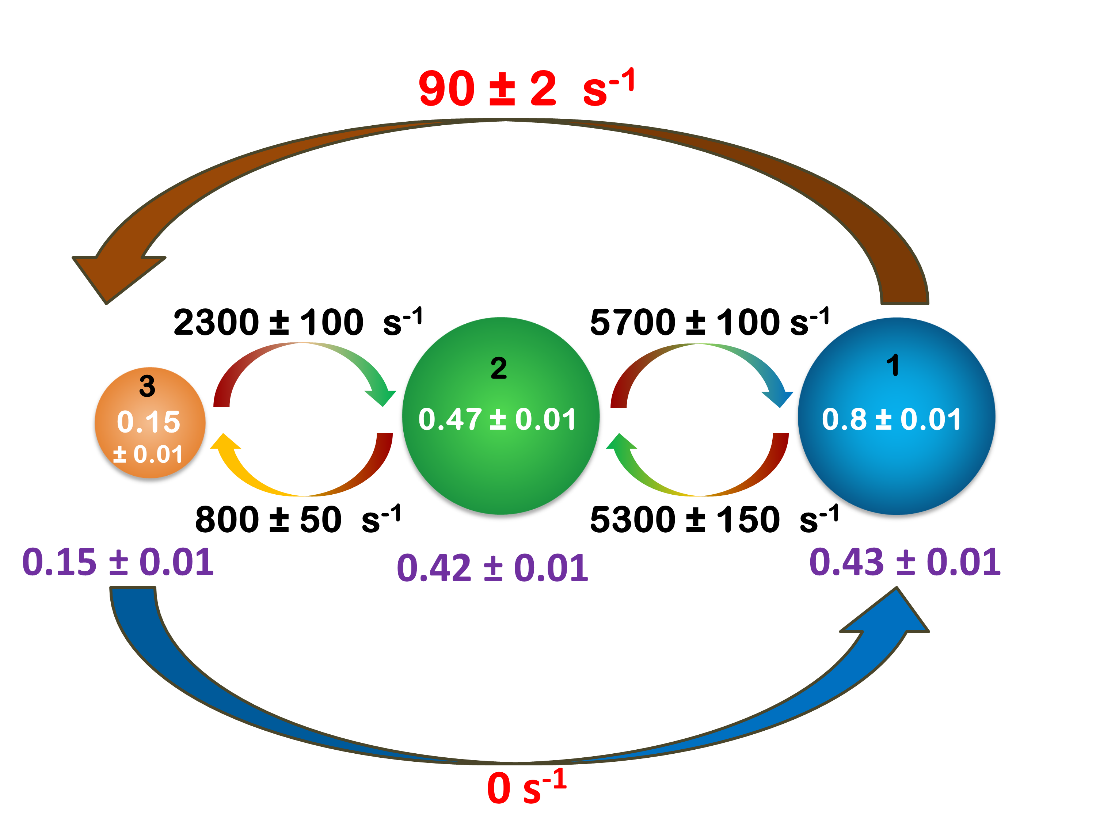


**Supplementary Figure 8. Full transition matrix as obtained from H^2^MM analysis.** smFRET data of ClpB molecules were analyzed using the H^2^MM algorithm, assuming three states. Three independent data sets were used. The analysis of each data set was initiated with 50 random initial guesses, spanning a broad range in parameter space. All analyses converged to an almost exactly the same model after hundreds of iterations. Here we show the full set of parameters obtained from the converged model of three independent data sets. The FRET efficiency values of the states are shown inside the circles in white, and state occupancies are shown below the circles in purple. The transition rates between states are given above or below the arrows. Clearly, the transitions involving the sequential pathway are much larger than other transitions, which can therefore be neglected.


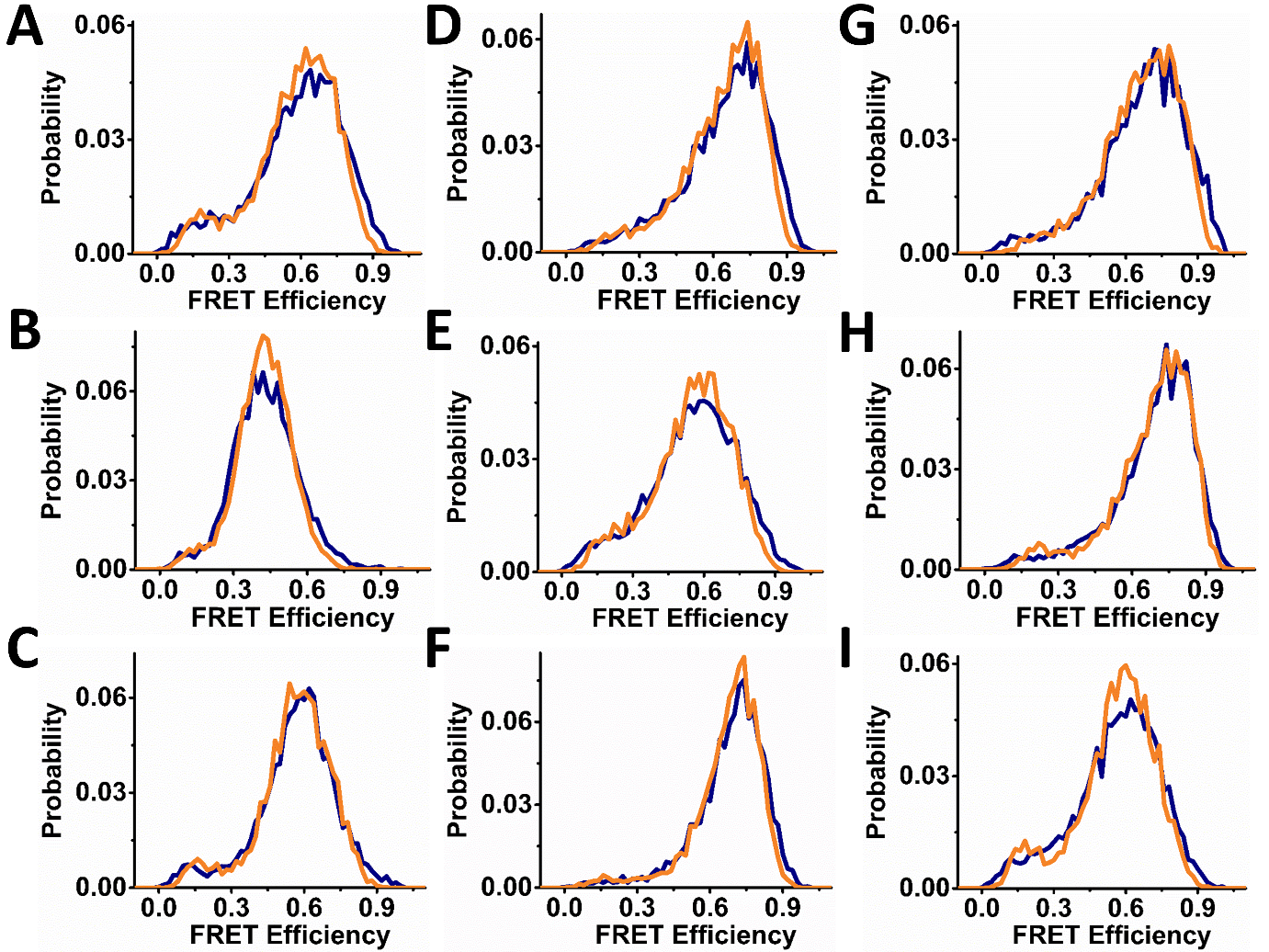


**Supplementary Figure 9. Line plots of FRET efficiency and recolored histograms.** Unsmoothed versions of all line plots of FRET histogram presented in the main text (Fig. 1 B, F, G, Fig. 3D and Fig. 5B) are shown in blue color. The orange lines are the outcomes of ‘recoloring’ simulations based on the H^2^MM analysis. (A) ClpB (S428-S771C), (B) ClpB (S359C-S428C), (C) ClpB (S359C-Q483C), (D) ClpB Walker A mutant [A^-^A^+^] (S428-S771C), (E) ClpB Walker A mutant [A^+^A^-^] (S428-S771C), (F) ClpB (S428C-S771C) with 25 µM DnaK, (G) ClpB (S428C-S771C) with 50 µM κ-Casein, (H) ClpB K347A (S428-S771C) and (I) ClpB E423A (S428-S771C). In all ClpB mutants and conditions, a three state model fits the data very well.


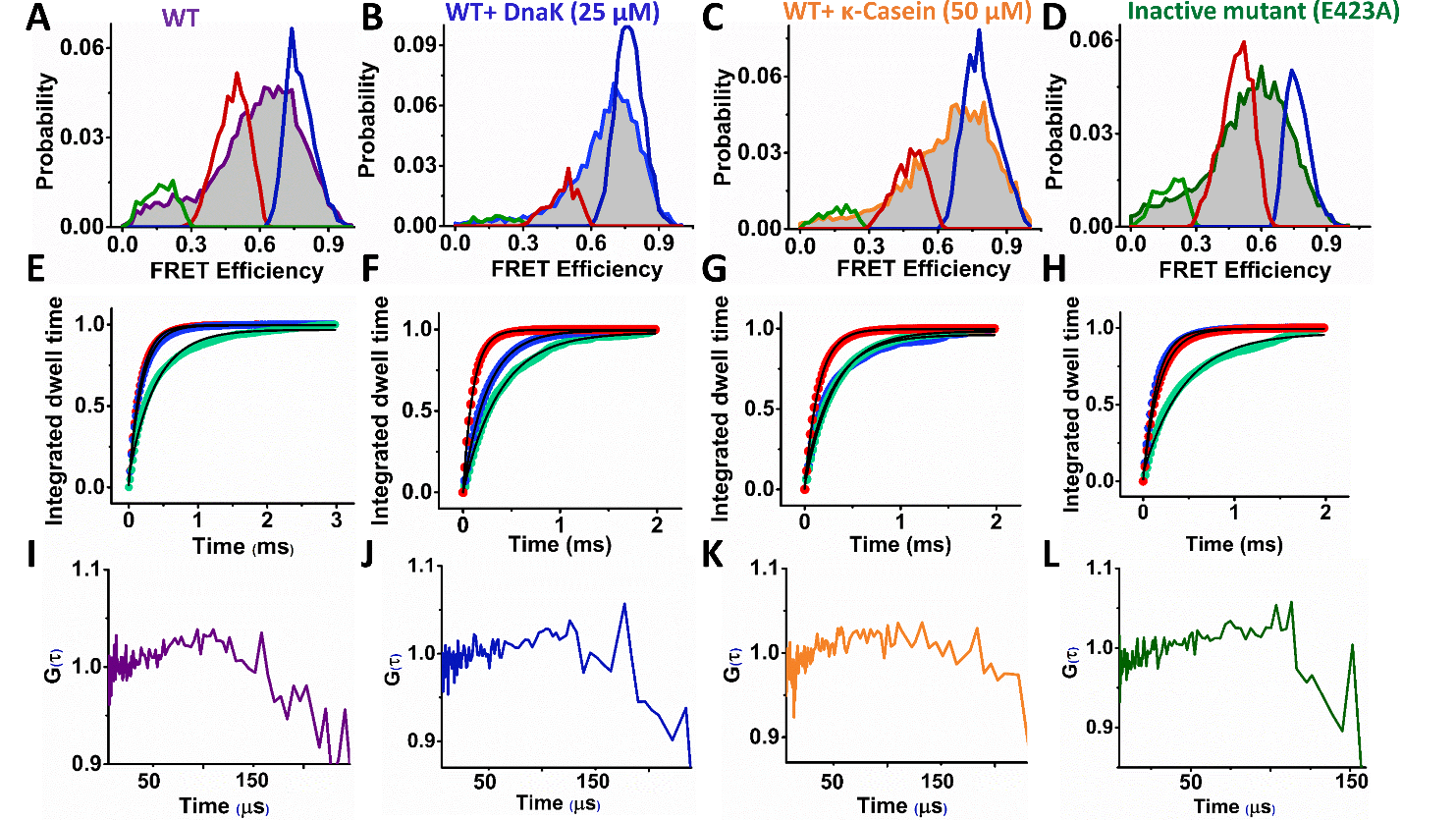


**Supplementary Figure 10. Verification of fast dynamics in smFRET data.** Verification analyses are shown for experiments performed under the conditions marked on top of each column. (A-D) Segmentation analysis of smFRET burst data based on the H^2^MM analysis parameters (Fig. 1 E). In this calculation we used the H^2^MM parameters and the Viterbi algorithm to assign segments of data in fluorescence bursts that belong to each of the three states. We then calculated the average FRET efficiency for each segment and plotted histograms of the segments belonging to each state ^18^. Segment-based FRET efficiency histograms showed a clear state separation. State 1 is shown in blue, state 2 in red and state 3 in green. (E-H) Integrated dwell-time distributions for the three states, calculated as described in ref. ^18^. State 1 is shown in blue, state 2 is shown in red and the third state is shown in green. Black lines are fits to single-exponential functions, and the obtained rates are listed in Supplementary Table 2. (I-L) Cross-correlation functions of the donor and acceptor fluorescence on a burst-by-burst basis^19^. The increase in the cross correlation at early times clearly indicates conformational dynamics.


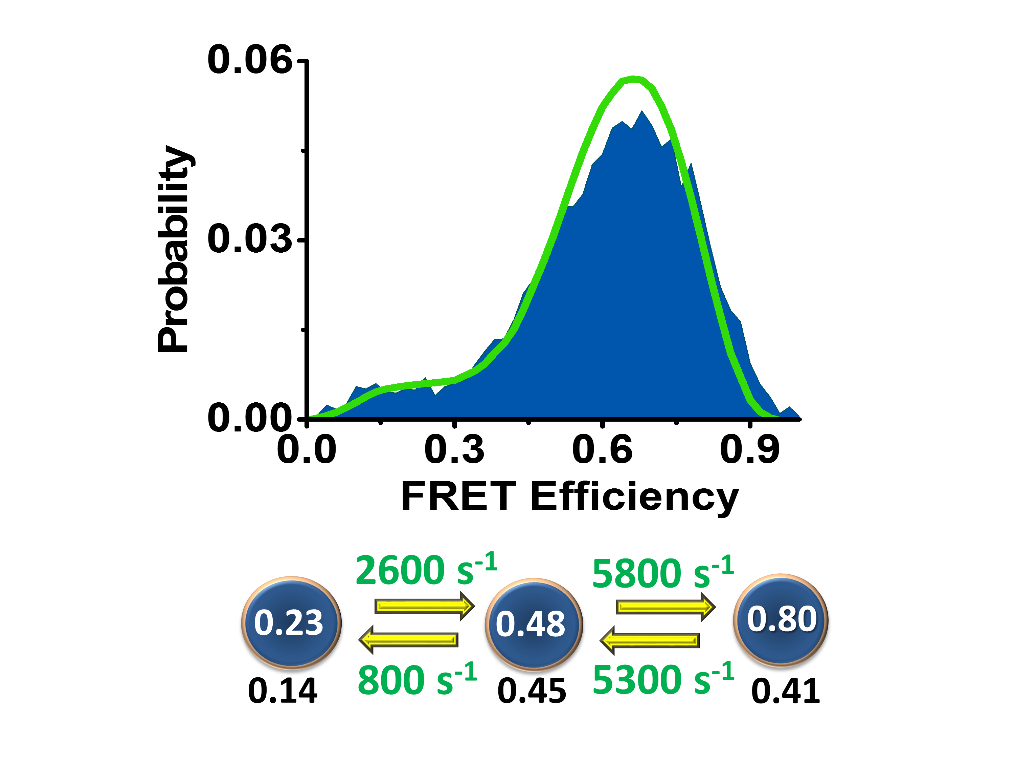


**Supplementary Figure 11. Effect of a non-hydrolysable nucleotide (ATPɣS) on the M-domain dynamics.** FRET efficiency histogram of *TT.* ClpB S428C-S771C in the presence of 2 mM ATPɣS. Recoloring based on a three-state model analysis with H^2^MM fits very well the histogram (green line). The FRET efficiency values of the three states (inside blue circles), their occupancy (below the circles) and the transition rates between them (above and below arrows) are very similar to those obtained in the presence of ATP (Fig. 1 E, main text). The errors on the rates are ± 150 s^-1^, as obtained from two repeats of the experiment. The errors on FRET efficiency values and state occupancies are similar to ClpB in the presence of ATP.


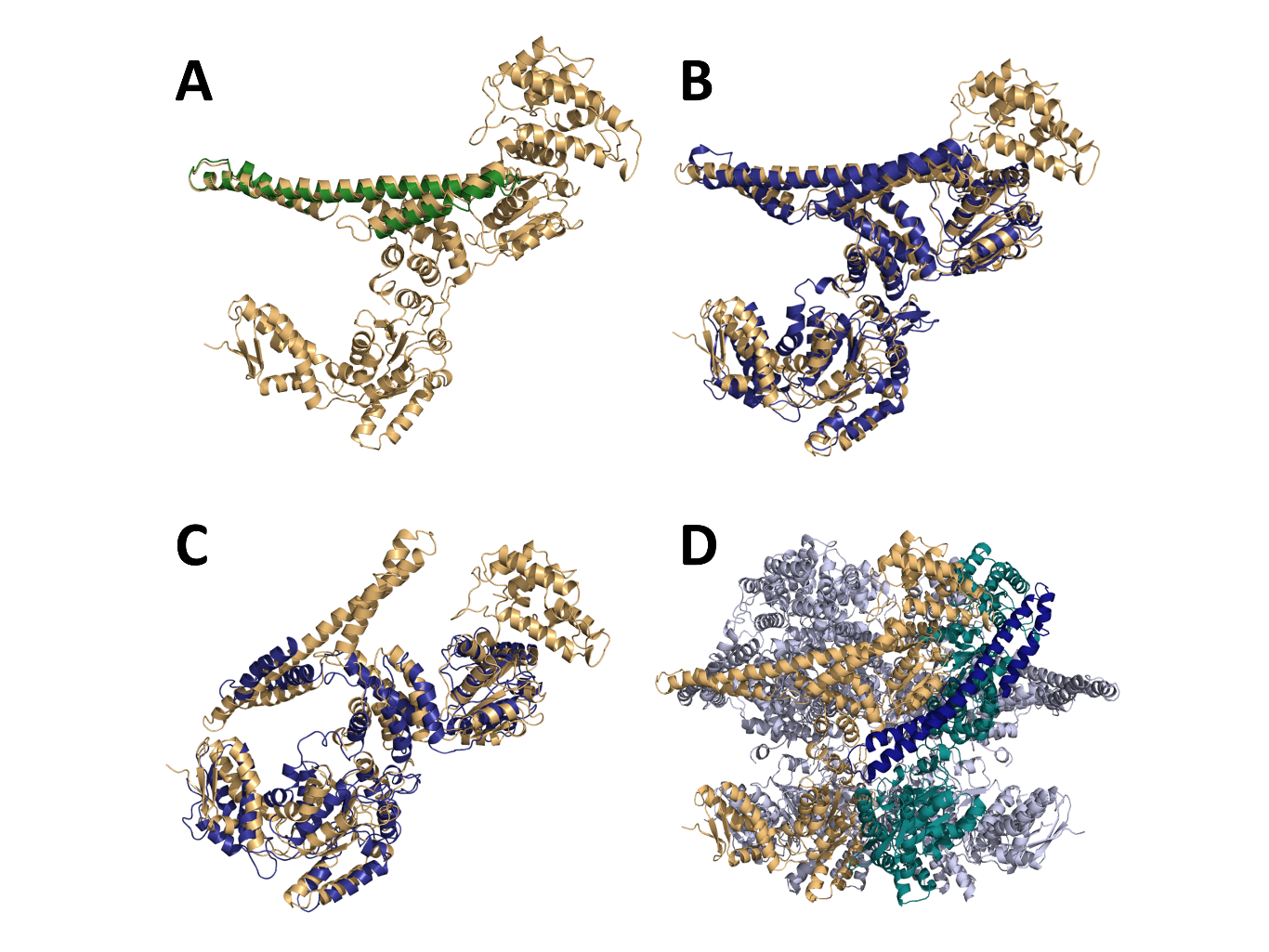


**Supplementary Figure 12. Comparison of the M-domain structural model to other models.** (A) Our model of ClpB with the M domain in the inactive conformation (shown in green), is compared with the crystal structure of the ClpB monomer (1QVR)^20^ shown in gold color. (B) The same model of ClpB with the M domain in the inactive conformation (now shown in gold) is compared with a recent cryo-EM model of ClpB from *E. coli* (5OFO)^21^ shown in dark blue. The two structures were aligned globally, using PyMol. (C) Our model of ClpB with the M domain in the active conformation (shown in gold color) is compared with a recent cryo-EM model of ClpB from E*. coli* (5OGO)^21^ shown in dark blue. Here also, the two structures were aligned globally, using PyMol. Note that the cryo-EM model contains only a partial structure of the M domain. (D) Tilted conformation of the M-domain (dark blue), showing that motif 1 is still close to the NBD1 of the adjacent protomer (light orange).


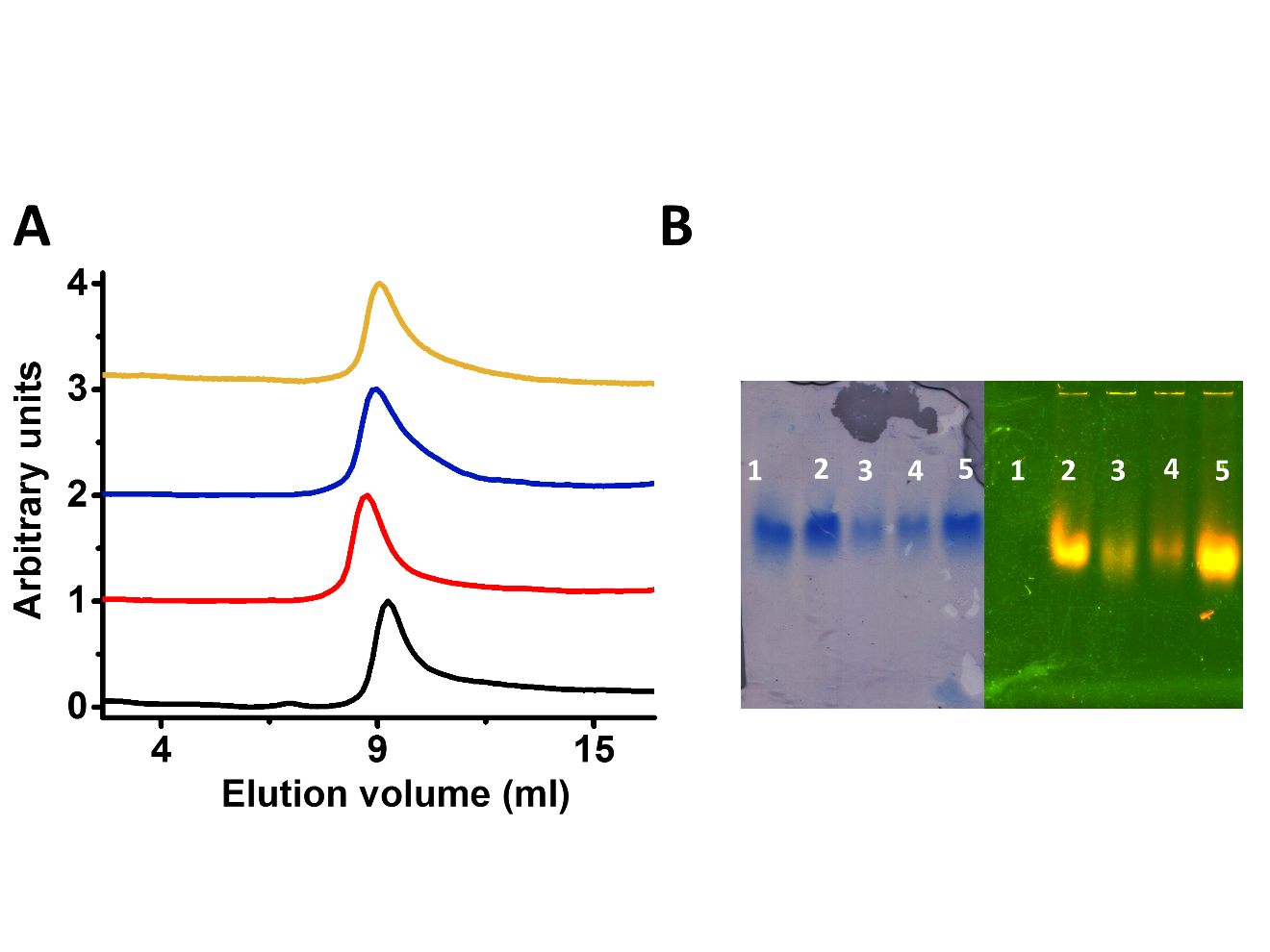


**Supplementary Figure 13. Characterizing the assembly of ClpB Walker mutants.** (A) Oligomerization state of ClpB Walker mutants was analyzed using gel filtration on a Superdex-200 column. The elution profile of WT ClpB and the various mutants are shown as follows: WT- black, [A^-^A^+^]- blue, [A^+^A^-^]- red and [B^-^B^-^]- orange. All Walker mutants used in this study showed almost the same elution profile as the WT, an indication of full assembly. The buffer used in this experiment is similar to the smFRET buffer (25 mM HEPES, 25 mM KCl, 10 mM MgCl_2_, 2 mM ATP). The elution signal was recorded at 290 nm, the peaks were normalized and shifted in the vertical direction for clarity. (B) Left: Native gel (3% Acrylamide) stained with Coomasie Blue, ran under the same conditions as described in Supplementary Figure 1D. (1) WT ClpB, (2) double-labeled ClpB S428C-S771C, (3) double-labeled [A^-^A^+^] mixed 1:100 with non-labeled [A^-^A^+^], (4) double-labeled [A^+^A^-^] mixed 1:100 with non-labeled [A^+^A^-^] and (5) 1 double-labeled [B^-^B^-^] mixed 1:100 with non-labeled [B^-^B^-^]. Right: same gel as in left part, imaged with a Typhoon scanner at 473 nm and 532 nm. The combination of the two scans presented yellow bands indicating double-labeled complexes. Both gels show single bands and similar migration as the WT, an indication of fully assembled complexes.


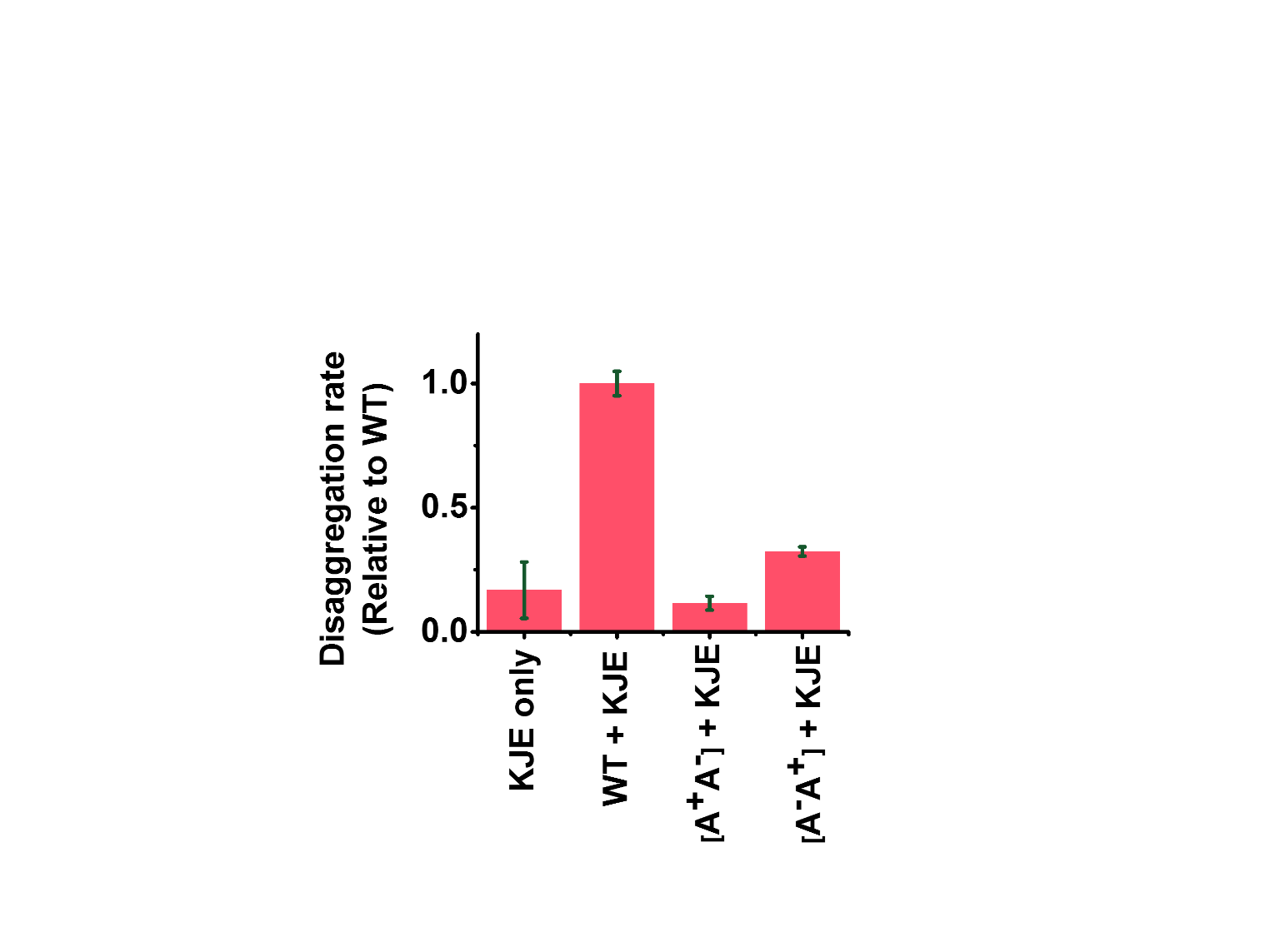


**Supplementary Figure 14. Luciferase disaggregation by ClpB.** 50 nM of pre-aggregated inactive luciferase was prepared as described in (ref. ^22^), but instead of GdmCl we used urea as a chemical denaturant. The inactive luciferase was incubated at 27^o^C in the presence of 5 mM ATP, 200 mM glycine betaine, 100 mM Tris-acetate at pH 7.5, 150 mM KCl, 10 mM MgCl_2_, 5 mM DTT and different combinations (as indicated) of 2 µM DnaK, 0.4 µM DnaJ, 1 µM GrpE and 1 µM WT ClpB or ClpB variants for a total time of 6 hrs. Activity of reactivated luciferase was measured at different time points of the reaction as follows: 2.5 µl from each reaction mix was transferred into 80 µl of a luciferase reaction mix (50 mM Tris-acetate pH 7.5, 15 mM MgCl_2_, 5 mM ATP, 50 mM KCl, 200 µM coenzyme A and 5 µM D-luciferin) in a white opaque plate. Luminescence intensity values were then recorded for 20 seconds. For each ClpB sample, we obtained a rate of disaggregation which was then normalized by the rate of WT ClpB disaggregation as shown in the figure. Error bars were obtained from three repeats of the experiment.


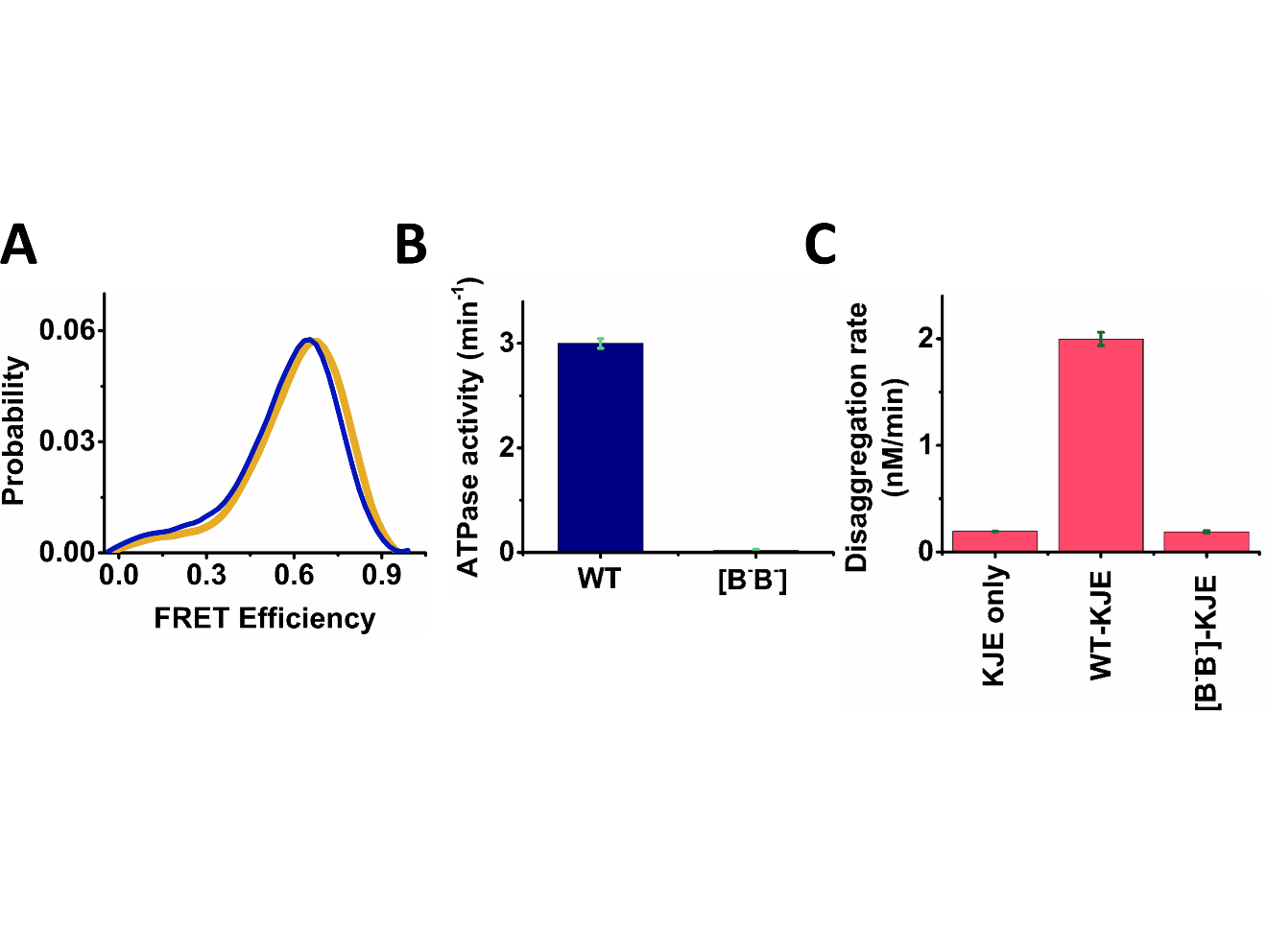


**Supplementary Figure 15. Effect of Walker B mutants on the M-domain dynamics.** ClpB double-Walker B mutant ([B^-^B^-^]) was generated, purified, labeled and assembled using similar procedures as described above. (A) FRET efficiency histogram of [B^-^B^-^] mutant in 2 mM ATP (dark blue line) is very similar to the histogram obtained with the WT ClpB (orange line). H^2^MM analysis shows also similar results to the WT analysis (Supplementary Tables 4-5). This indicates that it is not the perturbation due to mutation in the NBDs that causes the change in M-domain dynamics obtained with other Walker mutants. Rather, M-domain dynamics are modified due to the modulation of ATP binding in each NBD. (B – C) ATPase activity (in comparison to WT) and disaggregation activity show that [B^-^B^-^] is not active at all due to deficiency in ATP hydrolysis, as expected for Walker B mutants ^23,24^.


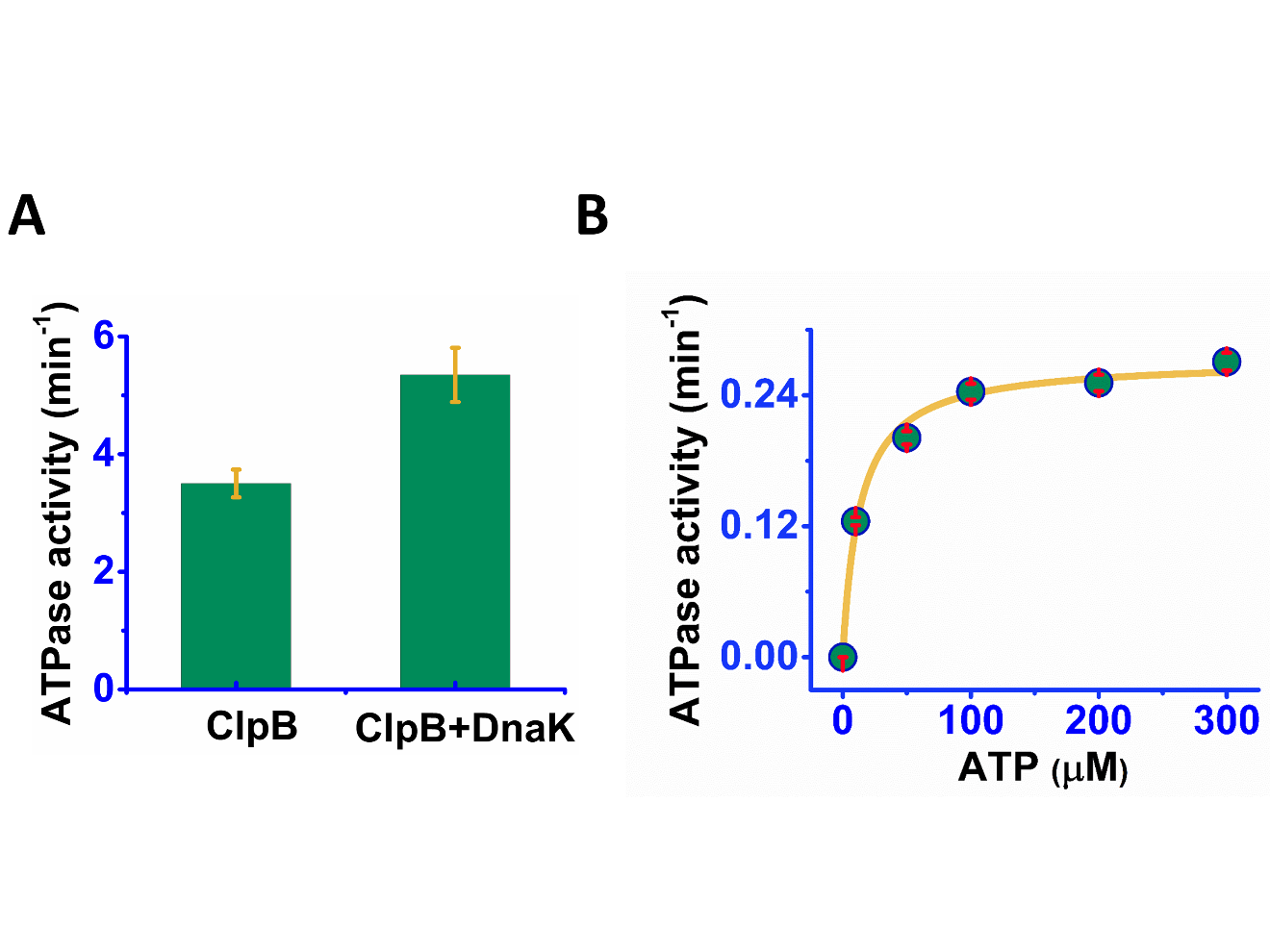


**Supplementary Figure 16. DnaK characterization.** (A) WT ClpB ATPase activity was measured without and with 2 µM DnaK and in the presence of 2 mM ATP. The stimulation of ATPase activity by DnaK indicates that it is folded properly. (B) DnaK ATPase activity was measured at 30 ^o^C, using the same methodology as for ClpB. A fit to the Michaelis - Menten equation yielded a rate constant of 0.27 ± 0.01 min^-1^, and a K_m_ value of 13.4 ± 2.2 µM, similar to a previous report ^25^.





**Supplementary Figure 17. Third state population as a function of DnaK concentration.** smFRET measurements of ClpB (S428C-S771C) in the presence of increasing concentrations of DnaK were analyzed using H^2^MM with the three-state model. Results show that the third state population is decreasing strongly with increasing DnaK concentration.





**Supplementary Figure 18. Dissociation constant of DnaK - ClpB estimated from disaggregation activity.** G6PDH aggregates preparation and disaggregation activity assays were conducted similarly to the protocol described previously (see Methods section). Here we used constant concentrations of ClpB (2 µM), DnaJ (1 µM) and GrpE (1 µM), but different concentrations of DnaK (0 – 60 µM). The rate of disaggregation at each DnaK concentration was measured similar to the description in the Methods (main text). The red line is a fit to the Hill equation (equation 3 in Methods, main text), yielding a K_0.5_ value of 5.7 ± 1 µM. The Hill coefficient obtained from the fit is 2.05 ± 0.18, which indicates that the minimum number of DnaK molecules bound to ClpB is 2.





**Supplementary Figure 19. Effect of κ-Casein binding in the presence of a non-hydrolysable nucleotide (ATPɣS) on the M-domain dynamics.** To test whether κ-Casein binding to ClpB in the presence of 2 mM ATPɣS can lock the M-domain in one conformation as indicated by Deville *et al ^21^*, we conducted smFRET measurement of ClpB (S428C - S771C) in the presence of 20 µM κ-Casein and 2 mM ATPɣS. The FRET efficiency histogram showed a broad FRET distribution that was very similar to the result obtained in the presence of 2 mM ATP (red line) (see Supplementary Table 8).


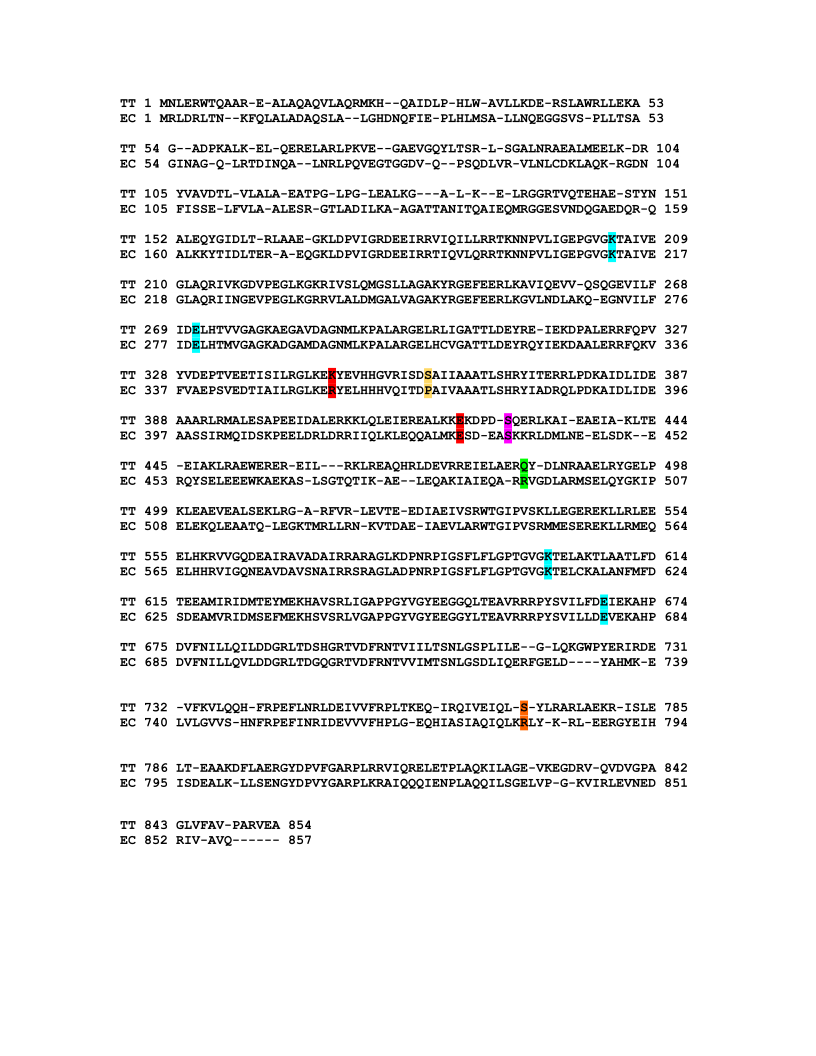


**Supplementary Figure 20. *Thermus thermophiles* (TT) ClpB sequence with marked mutation sites.** The figure shows sequence alignment of TT ClpB with *E. coli* ClpB variant. Walker A and Walker B mutants are marked in cyan, and red color marks active and inactive mutants. Labeling sites are marked as follows: yellow- S359C, pink- S428C, green- Q483C, orange- S771C. The alignment was done using Sequence Manipulation Suite, Pairwise Align Protein Tool, using scoring matrix BLOSUM 80.


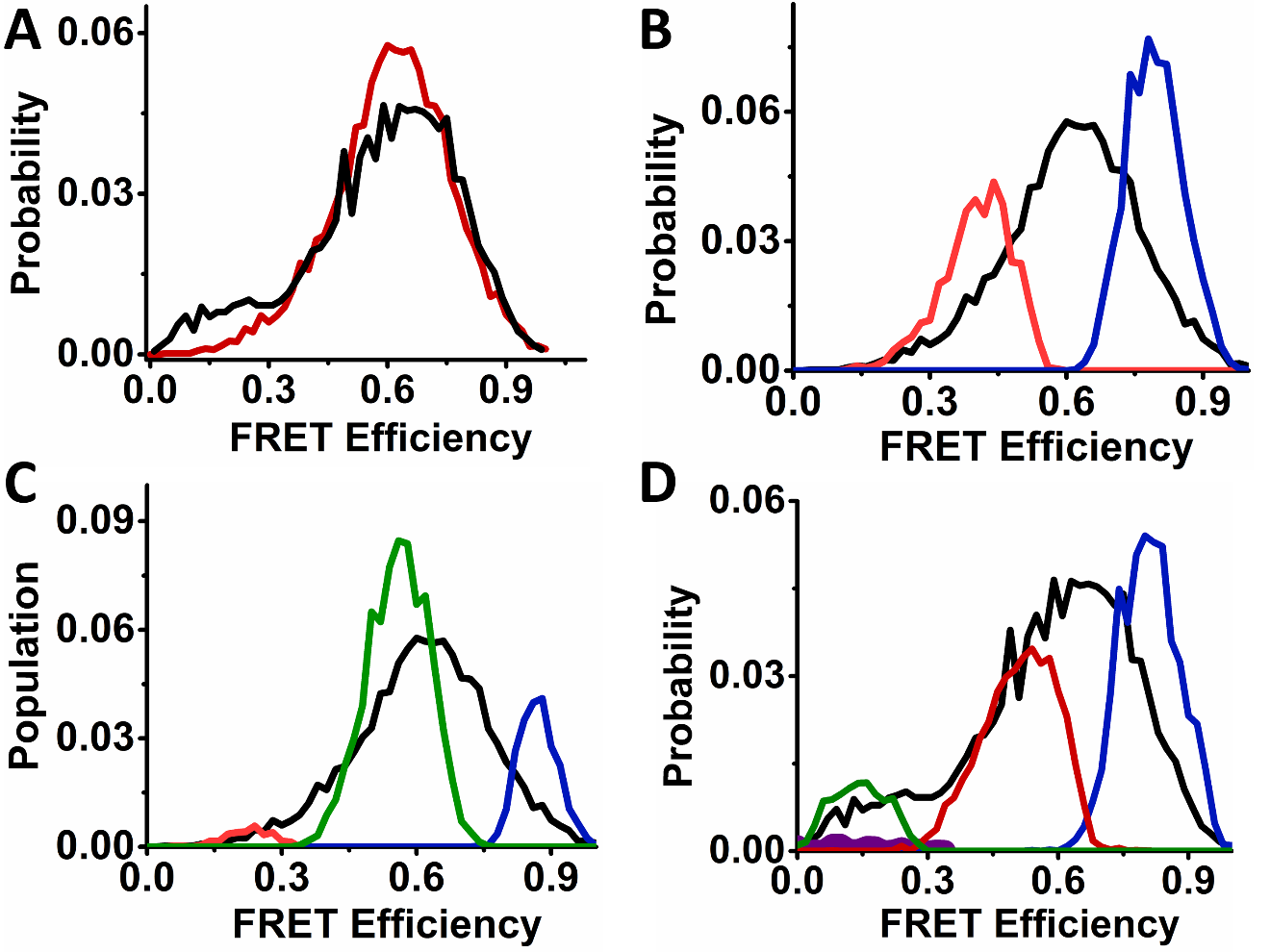


**Supplementary Figure 21. Testing the suitability of the three-state model.** To test the validity of representing of the main FRET peak in measured histogram with two states, we simulated data based on diffusion in a single harmonic potential well (red line), and compared it to our experimental data (black line). For details of the simulation and further analysis described below, see Supplementary Notes. (B) Segmentation analysis of the simulated data, based on H^2^MM analysis with a two state model. Black line is the simulated data, red line is state 1 and blue line is state 2. (C) Analysis with a three-state model leads to different positions for the segmented states than in B. Black line is the simulated data, red line is state 1, green line is state 2 and blue line is state 3. (D) Analysis of the experimental data with four states does not improve the solution, and provides exactly the same FRET states as with a three-state model. Black line is the real data, blue line is state 1, red line is state 2 and green line is state 3. The fourth state is shown in purple as a small population that overlaps with state 3.

**Supplementary Table 1. State-to-state transition rates (in s^-1^) between states obtained from three-state model analysis of three different ClpB FRET pairs**

| **ClpB variant** | **k_12_** | **k_21_** | **k_23_** | **K_32_** |
| --- | --- | --- | --- | --- |
| **S428C – S771C** | 5300 ± 150 | 5700 ± 100 | 800 ± 50 | 2300 ± 100 |
| **S359C - S428C** | 6900 ± 300 | 4900 ± 300 | 290 ± 150 | 1290 ± 100 |
| **Q483C-S359C** | 5390 ± 400 | 4100 ± 200 | 330 ± 20 | 990 ± 200 |

The errors were calculated from at least three independent repeats of the experiment.

**Supplementary Table 2. Verification of H^2^MM analysis with dwell time analysis of several experiments**

| **ClpB variant** | **State 1** | | **State 2** | | **State 3** | |
| --- | --- | --- | --- | --- | --- | --- |
|  | **H^2^MM** | **Dwell time** | **H^2^MM** | **Dwell time** | **H^2^MM** | **Dwell time** |
| **WT** | 5400 ± 100 | 5100 ± 50 | 6500 ± 150 | 5900 ± 50 | 2300 ± 100 | 2100 ± 50 |
| **[A^-^A^+^]** | 3500 ± 100 | 3500 ± 100 | 7300 ± 400 | 7200 ± 100 | 4100 ± 10 | 3900 ± 100 |
| **E423A** | 7000 ± 400 | 6700 ± 100 | 6200 ± 200 | 5600 ± 100 | 1685 ± 250 | 2200 ± 100 |
| **DnaK**  **(25 µM)** | 4300 ± 200 | 4000 ± 50 | 9300 ± 400 | 9450 ± 50 | 1450 ± 150 | 2600 ± 50 |
| **κ-Casein**  **(50 µM)** | 4800 ± 300 | 3900 ± 100 | 7000 ± 350 | 6800 ± 50 | 3100 ± 150 | 3200 ± 100 |

Comparison of rates (in s^-1^) from H^2^MM analysis with rates from integrated dwell-time distributions for each state of the M domain. The rates in the table are the sums of the rates of all transitions out of a given state. The errors were calculated from at least two independent repeats of the experiment.

**Supplementary Table 3.** **Comparison of the FRET efficiencies of state 1 and state 2 obtained from H^2^MM analysis and from the 10 simulated structures with the lowest chi-squared values**

| **ClpB variant** | **Inactive**  **χ^2^ = 0.060 ± 0.005*** | | **Active**  **χ^2^ = 0.0058 ± 0.0008** | |
| --- | --- | --- | --- | --- |
|  | **H^2^MM** | **Simulated structure**** | **H^2^MM** | **Simulated structure**** |
| **S428C - S771C** | 0.47 ± 0.02 | 0.61 ± 0.04 | 0.8 ± 0.02 | 0.85± 0.01 |
| **S359C - S428C** | 0.30 ± 0.02 | 0.34 ± 0.01 | 0.60 ± 0.02 | 0.57 ± 0.02 |
| **S359C - Q483C** | 0.75 ± 0.02 | 0.58 ± 0.03 | 0.47 ± 0.02 | 0.45 ± 0.01 |

* The average root mean squared deviation of the 10 simulated structures with the best chi-squared values and the crystal structure (PDB 1QVR) was 5.5 ± 3.1 Å. The root mean squared deviation of the simulated structure with the best chi-squared value was 1.2 Å.

**calculated from the 10 structures with the best chi-squared values.

**Supplementary Table 4. State-to-state transition rates (in s^-1^) obtained from H^2^MM** **analysis of ClpB mutants.**

| **ClpB variant** | **k_12_** | **k_21_** | **k_23_** | **K_32_** |
| --- | --- | --- | --- | --- |
| **WT** | 5300 ± 150 | 5700 ± 100 | 800 ± 50 | 2300 ± 100 |
| **K347A** | 3100 ± 200 | 5500 ± 200 | 670 ± 20 | 2100 ± 100 |
| **E423A** | 7000 ± 350 | 5600 ± 200 | 610 ± 100 | 1685 ± 200 |
| **[A^+^A^-^]** | 7700 ± 100 | 6000 ± 200 | 1500 ± 100 | 4100 ± 150 |
| **[A^-^A^+^]** | 3500 ± 100 | 5800 ± 350 | 1550 ± 350 | 4100 ± 10 |
| **[B^-^ B^-^]** | 5100 ± 150 | 6100 ± 150 | 750 ± 100 | 2500 ± 100 |

The errors were calculated from two independent repeats of the experiment.

**Supplementary Table 5. Relative population of state 1 and state 2 in ClpB WT and ClpB mutants**

| **ClpB variant** | **State 1 (active)** | **State 2 (inactive)** | **Ratio**  **(active/inactive)** |
| --- | --- | --- | --- |
| **WT** | 0.51 ± 0.01 | 0.49 ± 0.01 | 1.00 ± 0.01 |
| **K347A** | 0.62 ± 0.01 | 0.38 ± 0.01 | 1.63 ± 0.01 |
| **E423A** | 0.42 ± 0.01 | 0.58 ± 0.01 | 0.72 ± 0.01 |
| **[A^+^A^-^]** | 0.43 ± 0.02 | 0.57 ± 0.01 | 0.75 ± 0.02 |
| **[A^-^A^+^]** | 0.62 ± 0.02 | 0.38 ± 0.01 | 1.63 ± 0.02 |
| **[B^-^ B^-^]** | 0.51 ± 0.01 | 0.49 ± 0.01 | 1.00 ± 0.01 |

The relative populations of state 1 and state 2 were calculated from the three-state model as obtained from H^2^MM analysis, after excluding the third state population. The errors were calculated from at least two independent repeats of the experiment.

**Supplementary Table 6. Enzymatic activity parameters of ClpB Walker A mutants.**

| **ClpB variant** | **K_cat_ (1/min)** | **K_0.5_ (µM)** | **n** |
| --- | --- | --- | --- |
| **WT** | 3.2 ± 0.1 | 390 ± 15 | 3.1 |
| **[A^-^A^+^]** | 0.32 ± 0.01 | 320 ± 20 | 2 |
| **[A^+^A^-^]** | 0.1 ± 0.03 | 500 ± 73 | - |

Model parameters from fits of ATPase activity of WT ClpB and Walker mutants, obtained either from a fit to the Hill equation (WT and [A^-^A^+^], equation. 3 main text) or to the Michaelis-Menten equation ([A^+^A^-^]).

**Supplementary Table 7. Transition rates (in s^-1^) between the active and inactive M-domain conformation at different DnaK concentrations.**

| **DnaK (µM)** | **k_12_** | **k_21_** |
| --- | --- | --- |
| **0** | 5300 ± 150 | 5700 ± 100 |
| **5** | 5500 ± 150 | 7400 ± 450 |
| **25** | 4300 ± 200 | 9100 ± 350 |

The errors were calculated from at least three independent repeats of the experiment.

**Supplementary Table 8. State-to-state transition rates (in s^-1^) from experiments with different κ-Casein concentrations**

| **κ-casein (µM)** | **k_12_** | **k_21_** |
| --- | --- | --- |
| **0** | 5300 ± 150 | 5700 ± 100 |
| **10** | 5200 ± 200 | 5800 ± 150 |
| **20** | 5100 ± 300 | 6800 ± 350 |
| **50** | 5000 ± 200 | 7200 ± 350 |
| **100** | 5800 ± 100 | 7600 ± 100 |

The errors were calculated from at least two independent repeats of the experiment.

**Supplementary Table 9. Transition rates (in s^-1^) from experiments with different G6PDH aggregate concentrations.**

| **G6PDH aggregates**  **(nM)** | **k_12_** | **k_21_** |
| --- | --- | --- |
| **0** | 5300 ± 150 | 5700 ± 100 |
| **1** | 5600 ± 100 | 6600 ± 100 |
| **10** | 6600 ± 400 | 10900 ± 300 |
| **150** | 7900 ± 350 | 13300 ± 400 |

The errors were calculated from two independent repeats of the experiment.

**Supplementary Table 10. Relative population of state 1 and state 2 as a function of G6PDH aggregate concentration**

| **G6PDH aggregates**  **(nM)** | **State 1**  **(active)** | **State 2**  **(inactive)** | **Ratio**  **(active/inactive)** |
| --- | --- | --- | --- |
| **0** | 0.51 ± 0.01 | 0.49 ± 0.01 | 1.00 ± 0.01 |
| **1** | 0.53 ± 0.01 | 0.47 ± 0.01 | 1.13 ± 0.01 |
| **10** | 0.61 ± 0.01 | 0.39 ± 0.01 | 1.50 ± 0.01 |
| **150** | 0.63 ± 0.01 | 0.37 ± 0.01 | 1.70 ± 0.01 |

The relative populations of state 1 and state 2 were calculated from the three-state model as obtained from H^2^MM analysis, after excluding the third state population. The errors were calculated from at least two independent repeats of the experiments.

**Supplementary Table 11. Free-energy barriers (in K_B_T) for transitions between inactive and active states**

| **ClpB variant** | **ΔG^‡^ _12_** | **ΔG^‡^ _21_** |
| --- | --- | --- |
| **WT** | 2.90 ± 0.2 | 2.9 ± 0.2 |
| **K347A** | 3.5 ± 0.1 | 2.90 ± 0.2 |
| **E423A** | 2.6 ± 0.2 | 2.9 ± 0.2 |
| **[A^+^A^-^]** | 2.6 ± 0.1 | 2.8 ± 0.2 |
| **[A^-^A^+^]** | 3.6 ± 0.1 | 2.8 ± 0.2 |
| **[B^-^B^-^]** | 2.9 ± 0.2 | 2.8 ± 0.2 |

The free energy barriers were calculated from transition rates obtained from H^2^MM analyses, using the Arrhenius equation with a pre-exponential factor of 10^5^ s^-1^.

**Supplementary Table 12. List of primers of all ClpB mutants**

| **Name** | **Sequence** |
| --- | --- |
| **S359C** | 5’ gtgcgcatctccgac**tgc**gccatcatcgccgcc 3’ |
| **S428C** | 5’ gagaaggacccggac**tgc**caggagcgcctcaag 3’ |
| **Q483C** | 5’ ggagattgagctcgccgagcgg**tgc**tacgacctgaaccgggccgc 3’ |
| **N487C** | 5’ ccgagcggcagtacgacctg**tgc**cgggccgccgagctccgctac 3’ |
| **S771C** | 5’ gtggagatccagctc**tgc**tacctccgggcccgc 3’ |
| **K347A (active mutant)** | 5’ccggggcctcaaggag**gcg**tacgaggtccaccacg 3’ |
| **E423A (inactive mutant)** | 5’ gaggccctgaagaag**gcg**aaggacccggactcc 3’ |
| **K204T (Walker A NBD1)** | 5’ gagcccggcgtgggg**acg**acggccatcgtggag 3’ |
| **K601T (Walker A NBD2)** | 5’ cccacgggggtgggg**acg**acggagctcgccaag 3’ |
| **E271A (Walker B NBD1)** | 5’ catcctcttcattgac**gcg**ctccacaccgtggtg 3’ |
| **E668A (Walker B NBD2)** | 5’ gtcatcctctttgac**gcg**attgagaaggcccac 3’ |

**Supplementary Table 13. Photophysical properties of fluorescent dyes attached to ClpB**

| **Dye** | **Anisotropy** | **Quantum yield** | **Förster Radius (R_0_)**  **(nm)** |
| --- | --- | --- | --- |
| **Alexa 594 (S771C)** | 0.18 ± 0.01 | n.d. | 1. 55 ± 0.8 2. 54 ± 0.7 3. 53 ± 0.5 |
| **Alexa 488 (S428C)** | 0.16 ± 0.02 | 0.72 ± 0.06 |  |
| **Alexa 594 (S359C)** | 0.21 ± 0.01 | n.d. |  |
| **Alexa 488 (Q483C)** | 0.22 ± 0.01 | 0.62 ± 0.04 |  |

FRET pair (1) is S428C-S771C, (2) is S359C-S428C and (3) is S359C-Q483C.

**References**

1 Mastronarde, D. N. Automated electron microscope tomography using robust prediction of specimen movements. *J Struct Biol* **152**, 36-51, doi:10.1016/j.jsb.2005.07.007 (2005).

2 Zheng, S. Q. *et al.* MotionCor2: anisotropic correction of beam-induced motion for improved cryo-electron microscopy. *Nat Methods* **14**, 331-332, doi:10.1038/nmeth.4193 (2017).

3 Rohou, A. & Grigorieff, N. CTFFIND4: Fast and accurate defocus estimation from electron micrographs. *J Struct Biol* **192**, 216-221, doi:10.1016/j.jsb.2015.08.008 (2015).

4 Scheres, S. H. RELION: implementation of a Bayesian approach to cryo-EM structure determination. *J Struct Biol* **180**, 519-530, doi:10.1016/j.jsb.2012.09.006 (2012).

5 Aguado, A., Fernandez-Higuero, J. A., Cabrera, Y., Moro, F. & Muga, A. ClpB dynamics is driven by its ATPase cycle and regulated by the DnaK system and substrate proteins. *Biochem J* **466**, 561-570, doi:10.1042/BJ20141390 (2015).

6 Uchihashi, T. *et al.* Dynamic structural states of ClpB involved in its disaggregation function. *Nat Commun* **9**, 2147, doi:10.1038/s41467-018-04587-w (2018).

7 Werbeck, N. D., Schlee, S. & Reinstein, J. Coupling and dynamics of subunits in the hexameric AAA+ chaperone ClpB. *J Mol Biol* **378**, 178-190, doi:10.1016/j.jmb.2008.02.026 (2008).

8 Kim, K. I. *et al.* Heptameric ring structure of the heat-shock protein ClpB, a protein-activated ATPase in Escherichia coli. *J Mol Biol* **303**, 655-666, doi:10.1006/jmbi.2000.4165 (2000).

9 Watanabe, Y. H., Motohashi, K. & Yoshida, M. Roles of the two ATP binding sites of ClpB from Thermus thermophilus. *J Biol Chem* **277**, 5804-5809, doi:10.1074/jbc.M109349200 (2002).

10 Akoev, V., Gogol, E. P., Barnett, M. E. & Zolkiewski, M. Nucleotide-induced switch in oligomerization of the AAA+ ATPase ClpB. *Protein Sci* **13**, 567-574, doi:10.1110/ps.03422604 (2004).

11 Woo, K. M., Kim, K. I., Goldberg, A. L., Ha, D. B. & Chung, C. H. The Heat-Shock Protein Clpb in Escherichia-Coli Is a Protein-Activated Atpase. *Journal of Biological Chemistry* **267**, 20429-20434 (1992).

12 Strub, C., Schlieker, C., Bukau, B. & Mogk, A. Poly-L-lysine enhances the protein disaggregation activity of ClpB. *FEBS Letters* **553**, 125-130, doi:10.1016/s0014-5793(03)00985-2 (2003).

13 Schlee, S. & Reinstein, J. The DnaK/ClpB chaperone system from Thermus thermophilus. *Cellular and Molecular Life Sciences* **59**, 1598-1606, doi:10.1007/pl00012486 (2002).

14 Muschielok, A. *et al.* A nano-positioning system for macromolecular structural analysis. *Nat Methods* **5**, 965-971, doi:10.1038/nmeth.1259 (2008).

15 Muschielok, A. & Michaelis, J. Application of the nano-positioning system to the analysis of fluorescence resonance energy transfer networks. *J Phys Chem B* **115**, 11927-11937, doi:10.1021/jp2060377 (2011).

16 Kalinin, S. *et al.* A toolkit and benchmark study for FRET-restrained high-precision structural modeling. *Nat Methods* **9**, 1218-1225, doi:10.1038/nmeth.2222 (2012).

17 Schlee, S., Groemping, Y., Herde, P., Seidel, R. & Reinstein, J. The chaperone function of ClpB from Thermus thermophilus depends on allosteric interactions of its two ATP-binding sites. *J Mol Biol* **306**, 889-899, doi:10.1006/jmbi.2001.4455 (2001).

18 Aviram, H. Y. *et al.* Direct observation of ultrafast large-scale dynamics of an enzyme under turnover conditions. *Proc Natl Acad Sci U S A* **115**, 3243-3248, doi:10.1073/pnas.1720448115 (2018).

19 Wahl, M., Gregor, I., Patting, M. & Enderlein, J. Fast calculation of fluorescence correlation data with asynchronous time-correlated single-photon counting. *Opt. Express* **11**, 3583-3591, doi:10.1364/OE.11.003583 (2003).

20 Lee, S. *et al.* The Structure of ClpB. *Cell* **115**, 229-240, doi:10.1016/s0092-8674(03)00807-9 (2003).

21 Deville, C. *et al.* Structural pathway of regulated substrate transfer and threading through an Hsp100 disaggregase. *Sci Adv* **3**, e1701726, doi:10.1126/sciadv.1701726 (2017).

22 Shorter, J. The mammalian disaggregase machinery: Hsp110 synergizes with Hsp70 and Hsp40 to catalyze protein disaggregation and reactivation in a cell-free system. *PLoS One* **6**, e26319, doi:10.1371/journal.pone.0026319 (2011).

23 Hoskins, J. R., Doyle, S. M. & Wickner, S. Coupling ATP utilization to protein remodeling by ClpB, a hexameric AAA+ protein. *Proc Natl Acad Sci U S A* **106**, 22233-22238, doi:10.1073/pnas.0911937106 (2009).

24 Hodson, S., Marshall, J. J. & Burston, S. G. Mapping the road to recovery: the ClpB/Hsp104 molecular chaperone. *J Struct Biol* **179**, 161-171, doi:10.1016/j.jsb.2012.05.015 (2012).

25 Palleros, D. R., Reid, K. L., Shi, L. & Fink, A. L. DnaK ATPase activity revisited. *FEBS Letters* **336**, 124-128, doi:doi:10.1016/0014-5793(93)81624-9 (1993).
